# Nocturnal Risk Assessment and Its Association With Anxiety Symptoms

**DOI:** 10.1111/psyp.70275

**Published:** 2026-03-23

**Authors:** Derek P. Spangler, Richa Gautam, Jennifer T. Kubota, Jasmin Cloutier, Nina Lauharatanahirun

**Affiliations:** ^1^ Department of Biobehavioral Health The Pennsylvania State University University Park Pennsylvania USA; ^2^ Department of Psychological and Brain Sciences University of Delaware Newark Delaware USA; ^3^ Department of Political Science and International Relations University of Delaware Newark Delaware USA; ^4^ Department of Biomedical Engineering The Pennsylvania State University University Park Pennsylvania USA

## Abstract

Human risk assessment (RA), the attentional and behavioral activities involved in detecting and analyzing threat, is feasibly enhanced at night to protect against hidden danger. However, this enhanced RA at night might come at a cost and thus signal increased risk for anxiety disorders. To test the hypothesis that the nighttime enhances RA and its associations with anxiety, the current study randomly assigned healthy volunteers (*N* = 87, Mean age = 22.4; 69% Female) to visit the laboratory at day or night. Participants completed a novel task that presented images depicting neutral content, injury threat (e.g., weapon) or infection threat (e.g., person sneezing). Each image was followed by a lottery decision. The task estimated RA's attentional component as threat‐induced bradycardia—cardiac deceleration to threat images. RA's behavioral component was estimated as threat‐induced risk aversion—decrease in risky choice following threat images. Anxiety symptoms were self‐reported on the Depression, Anxiety, and Stress Scale (DASS‐21). The cardiac results partially supported the “nocturnal enhancement” hypothesis: (1) The night but not the day group exhibited a sustained, non‐habituating pattern of threat‐induced bradycardia to infection threat images. (2) In the night but not the day group, individuals with higher bradycardia to infection images had a higher likelihood of elevated anxiety. Contrary to predictions, bradycardia to injury threat and risk aversion metrics, as well as their relations to anxiety symptoms, were not higher at night. Overall, time‐of‐day is shown to be an important variable to consider in studies of human threat responses and anxiety disorder risk. Based on our findings, the intuitive idea that humans have elevated RA at night is not straightforward; instead, the nighttime may selectively activate attentional orienting (vis‐à‐vis bradycardia) to ambiguous stimuli like those signaling risk of illness, with these nocturnal enhancements being subject to individual differences in anxiety.

## Impact

The current study provides novel evidence that the night period amplifies both: (i) cardiac deceleration towards image‐based threat and (ii) the inter‐person association between such cardiac decelerations and anxiety symptoms. Situating threat responses in an evolutionary‐ecological framework, findings suggest that time‐of‐day modulates cardiac threat responding and the expression of an anxiety phenotype related to autonomic/attentional functioning.

## 1 Introduction

Imagine you hear an unexpected noise at home alone. You might feel more scared if the noise was heard at night than during the day. The nighttime is profoundly connected to danger for diurnal creatures (Edensor 2015; Williams 2008) since nocturnal darkness conceals harmful agents (e.g., predators), making threats uncertain (Darwin 1871; Herrmann 2015; Nasar and Fisher 1993). Humans like other animals use *risk assessment* (RA) to mount a strategic defensive action against such uncertain and distal threats (Blanchard et al. 2011; Mobbs et al. 2020). RA is the “pattern of activities involved in the detection and analysis of threat stimuli and the situations in which the threat is encountered” (Blanchard et al. 2011) and involves attentional and behavioral components (Blanchard and Blanchard 2008). In the attentional component, we gather information about the threat to detect and perceive its features (Blanchard 2018). In the behavioral component, we choose cautious actions that minimize exposure to danger (Blanchard and Blanchard 2008, 1989). RA functions to reduce ambiguity about the threat and guide defensive actions that optimize survival. Since the nighttime witnesses heightened threat responses, the nighttime might also be associated with enhanced RA.

RA's components manifest at different stages of the *defense cascade* (Fanselow 1994), where defense becomes more active as spatiotemporal distance from the threat decreases. At the distant pre‐encounter stage, RA emerges in an anxiogenic context before threat detection (Mobbs et al. 2020; Moscarello and Penzo 2022; Tseng et al. 2023). Pre‐encounter RA involves vigilance: the animal suppresses ongoing behavior and scans the environment for danger in a sustained, non‐specific manner (Oken et al. 2006; Beauchamp 2015; Blanchard, Blanchard, Rodgers, and Weiss 1990; Blanchard, Blanchard, Tom, and Rodgers 1990). At the post‐encounter stage, RA involves orienting to a distal threat (Blanchard et al. 2011; Mobbs et al. 2015): the animal inhibits behavior and attends to the novel stimulus in a transient, specific manner (Sokolov 1960). Post‐encounter RA also involves behavioral caution and a suppression of reward‐seeking after orienting (Blanchard, Blanchard, Rodgers, and Weiss 1990; Blanchard, Blanchard, Tom, and Rodgers 1990; Mobbs and Kim 2015). Both forms of RA work together to enhance survival. Vigilance amplifies orienting responses to threats and blunts their habituation across repeated threats (Kastner‐Dorn et al. 2018; Zukerman et al. 2019; McCurry et al. 2024). Blunted habituation in post‐encounter responses may reflect vigilance since habituation and vigilance are both distributed across time (Oken et al. 2006; Mackworth 1964). Post‐encounter RA can be elicited in humans by asking them to view threat‐depicting photographs, representing distal threats in the defense cascade (Lang et al. 2000).

The attentional component of (post‐encounter) RA can be estimated with *threat‐induced bradycardia*—the transient slowing of heartbeats when registering an aversive stimulus such as a threat‐depicting image (Battaglia et al. 2024; Campbell et al. 1997; Lang et al. 1997). Sometimes termed “fear bradycardia” (Campbell et al. 1997) or “freezing,” (Roelofs and Dayan 2022), threat‐induced bradycardia likely reflects an orienting response to novel, salient information across species (Bradley 2009). Supporting this account, unpleasant versus neutral images evoke stronger bradycardia alongside greater pupil dilation (Hermans et al. 2013; Bradley 2009) and brain activity suggestive of heightened perception (Lang and Bradley 2010). We view threat‐induced bradycardia as a probabilistic estimator of orienting to threat such that stronger bradycardia suggests greater attentional engagement coinciding with immobility. Threat‐induced bradycardia may be a risk factor for anxiety (Battaglia et al. 2023; Schipper et al. 2019). Threat‐induced bradycardia has an elevated magnitude and blunted habituation in anxious individuals and anxiogenic contexts (Thayer et al. 2000; Stegmann et al. 2024; Chalmers et al. 1975).

RA's behavioral component can be estimated with *threat‐induced risk aversion—*cautious decision‐making in which choices become less risky after exposure to aversive stimuli (Clark et al. 2012; Kasheer and Nam 2021; Carr and Steele 2010). RA is a “neuroeconomic process” (McNaughton and Corr 2018): The animal becomes sensitive to situational uncertainty, leading to risk‐averse behavior that enhances survival (Crane et al. 2024; Mobbs et al. 2020). This involves reducing reward‐seeking (e.g., foraging) when threats/rewards are uncertain, allowing the animal to focus its energetic resources on monitoring threat (Blanchard and Blanchard 2008, 1989). Risk aversion can be indexed as a behavioral preference in economic lottery tasks (Holt and Laury 2002). Decisions are made about investing money in one of two lottery options where one option is more uncertain. A participant is risk‐averse if they choose fewer options with uncertain outcomes (Holt and Laury 2014). There are two types of risk situations where the outcome probabilities of a choice are known (*risk with no ambiguity*) or unknown (*risk with ambiguity*) (Lauharatanahirun et al. 2025). Choices, their neural correlates, (Hsu et al. 2005; Krain et al. 2006), and possibly RA (McNaughton and Corr 2018), differ between the two risk situations.

Evolutionarily, human survival would have benefited from enhanced RA during waking night hours to better cope with uncertain threats (e.g., predators) cloaked in darkness (Wichlinski 2022). The nocturnal period's enhancement of RA likely involves heightened average RA (elevated RA) as well as blunted habituation in RA responses across threat exposures (sustained RA; Stankowich and Blumstein 2005; Herry et al. 2007; Grissom and Bhatnagar 2009; Vila et al. 2007). A state of attention/caution that is elevated and sustained across stimuli should enhance the detection/avoidance of uncertain threats at night (Wichlinski 2022; Blanchard, Blanchard, Rodgers, and Weiss 1990; Blanchard, Blanchard, Tom, and Rodgers 1990; Bell et al. 2009; Dalmaijer et al. 2021). In the defense cascade, the nocturnal setting may increase vigilance before threat detection, leading to enhanced RA to specific threat stimuli. We theorize that nocturnal enhancement of RA is a hard‐wired heuristic formed around diurnal animals' evolutionary adaptive environment (Cosmides and Tooby 2011; Wichlinski 2022) in which the night imperfectly signals darkness and uncertainty about environmental threats (Jedon et al. 2025; Bianco et al. 2025; Coss 2021; Yorzinski and Platt 2011; Nasar and Jones 1997). This nocturnal heuristic, like other evolutionary biases (Korteling and Toet 2020), likely arises in modern humans, even in indoor and illuminated environments. Such nocturnal RA may be driven by an endogenous circadian rhythm, where the suprachiasmatic nucleus (SCN) helps generate diurnal variation in threat‐related neural activity (e.g., amygdala; Koch et al. 2017; Buijs et al. 2003).

Nocturnal enhancement of RA in humans has not been clearly tested, although prior studies hint at such effects. The nighttime and darkness witness increased human threat responses including subjective fear, amygdala activation, and physiological reactivity (e.g., Emens et al. 2020; Li et al. 2015; McGlashan et al. 2021). Humans exhibit stronger eyeblink startle and cardiac deceleration to loud noise and stronger cardiac acceleration to threat images/sounds at night (Miller and Gronfier 2006; Pace‐Schott et al. 2014; Li et al. 2015). Those nocturnal responses emerge in lighted, indoor environments, consistent with “nocturnal enhancement of defense” being endogenous. Those threat responses are not necessarily indicative of RA; they are evoked by immediate threat (loud, inescapable noise) or reflect active fight/flight as opposed to passive RA (cardiac acceleration signals active defense) (Sokolov and Cacioppo 1997). Precise measures of RA should capture its multimodal processes towards distal threats, such as images, which are more likely to elicit RA (McNaughton and Corr 2004).

Threat responses at night may promote survival, but if exaggerated, they could signal risk for anxiety disorders. The nocturnal setting, an evolutionarily salient threat context, is postulated to activate the RA processes at the core of anxiety symptoms (Blanchard et al. 2001). If so, then RA at night may represent a novel, evolutionarily rooted predictor of anxiety risk beyond traditional RA measures during the day. Hinting at such effects, darkness‐related startle responding is stronger in PTSD patients relative to controls (Grillon et al. 1998). Nocturnal modulation of RA‐anxiety links would expand on the traditional view of heightened RA as an anxiety disorder phenotype. This phenotype is possibly context‐dependent, such that the link between RA and anxiety risk is stronger at night versus day. Nocturnal enhancement of RA‐anxiety links is plausible since anxiety and the nighttime putatively increase vigilance (Davis and Whalen 2000; Wichlinski 2022), which may enhance post‐encounter RA. If such nocturnal enhancement is valid, then the pattern of greater threat‐induced bradycardia and risk aversion in high‐ versus low‐anxiety persons should be more prominent at night.

Injury threats such as predators are prototypical dangers in our evolutionary history (Blanchard et al. 2011). Therefore, nocturnal enhancement of RA and its links to anxiety might be stronger when RA is elicited by injury threats. However, *infection threats*, which signal exposure to pathogens, also require defense (Tybur and Lieberman 2016; Curtis et al. 2011; Schaller 2016; Bradley et al. 2001). Injury and infection threats rely on distinct neurocognitive architectures and emotional repertoires (fear versus disgust) (Stark et al. 2007; Schaller 2016). Infection threats may also elicit higher RA relative to injury threats, since sickness‐causing agents are microscopic and more ambiguous than more discernible injury threats (e.g., attacking dog; Schaller 2016). Indeed, infection threats elicit greater bradycardia (Gilchrist et al. 2016; Cisler et al. 2009), attentional capture (Cisler et al. 2009; Van Hooff et al. 2013) and event‐related potentials indicating heightened perception (Carretié et al. 2010; Mendoza‐Medialdea and Ruiz‐Padial 2022). Infection threats possibly required even stronger RA in our evolutionary nocturnal environment where darkness further concealed stimuli; in modern humans, this might lead to stronger nocturnal enhancement of RA towards infection versus injury threats.

**FIGURE 1:**
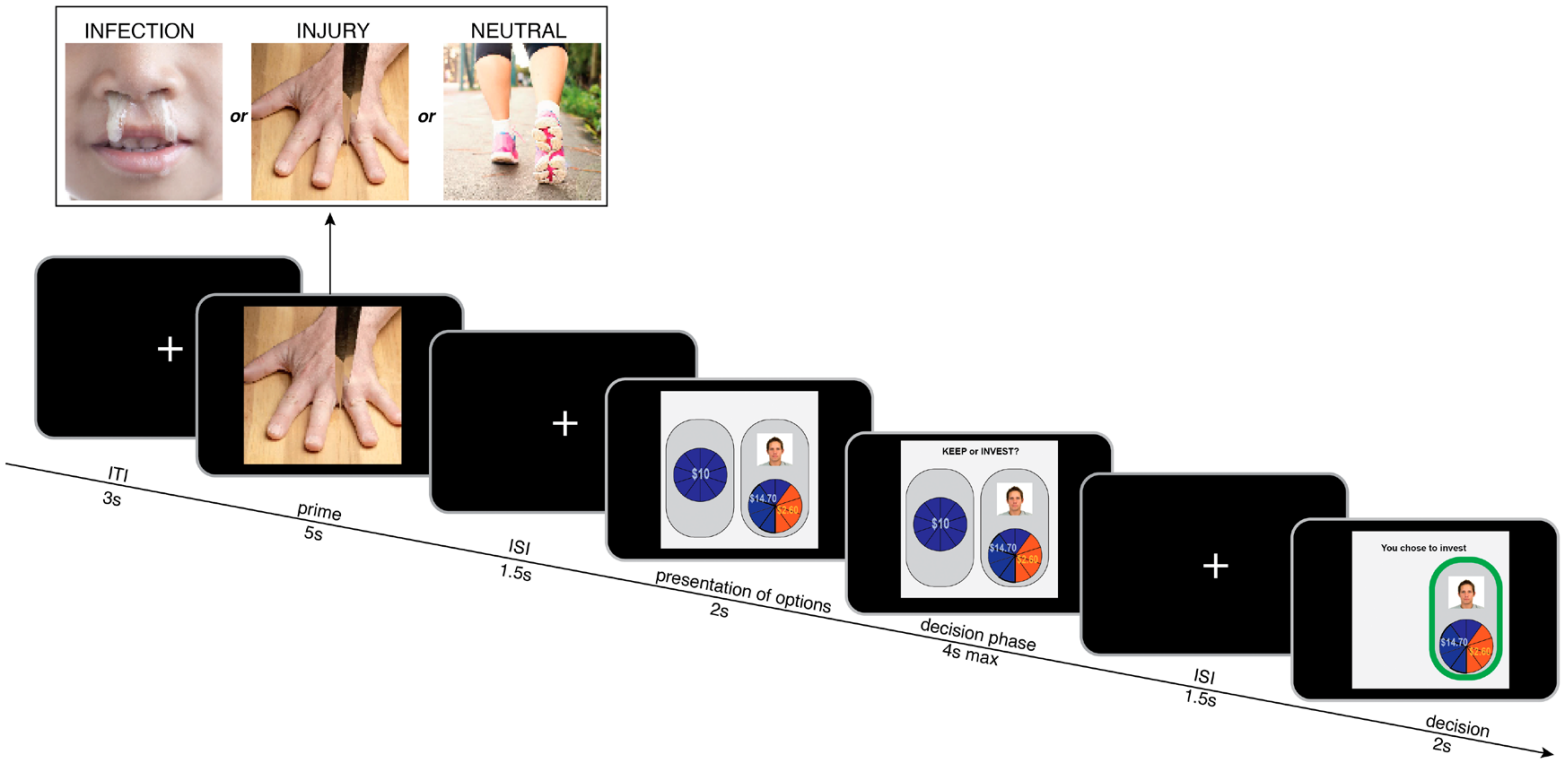
Schematic of a typical trial within the Risk Assessment Task. The task was used to induce and measure physiological and behavioral indices of risk assessment. Participants were presented with an image that varied in threat content (injury threat, infection threat, or neutral). Cardiac deceleration (bradycardia) was measured during the image presentation. Following the image, participants were asked to choose between either (1) keeping their endowment that ranged between $5 and $15 or (2) investing their endowment for the chance to earn potentially more money from either a social partner or non‐social probabilistic mechanism (i.e., roulette wheel).

The current study tested whether the nocturnal period enhances RA's attentional and behavioral components, estimated with threat‐induced bradycardia and threat‐induced risk aversion. RA was elicited in an image‐decision task that had two separate periods in each trial (Figure 1): (1) An image viewing period involved perception of threat images, which aimed to evoke RA throughout the trial. Image content was manipulated across three conditions: *neutral*, *threat of injury*, and *threat of infection* images. Threat‐induced bradycardia unfolded during image viewing. It was measured as RR interval lengthening during threat images (relative to neutral images), estimating the degree of attentional orienting. (2) A subsequent decisional period had participants make a two‐choice lottery decision. This period captured threat‐induced risk aversion, or the degree to which safe versus risky choice was primed by the earlier threat (versus neutral) image, providing an estimate of behavioral caution. Nocturnal effects on RA measures were examined by randomly assigning participants to a day or night session. To examine nocturnal RA as a correlate of anxiety risk, anxiety symptoms were reported on the Depression Anxiety and Stress scale (Antony et al. 1998). It was hypothesized that the nocturnal period would enhance RA measures—threat‐induced bradycardia and threat‐induced risk aversion—leading to stronger average levels and weakened habituation in these metrics. It was also predicted that the nocturnal period would amplify the inter‐person associations between RA measures and anxiety symptoms. We hypothesized that the aforementioned “nocturnal enhancement” effects would emerge for (i) RA to injury threat and (ii) RA to infection threat images. The current study also explored two questions. First, we explored whether nocturnal effects were stronger on RA towards injury versus infection threat images, given the scarcity of research on the topic. Second, we explored whether nocturnal effects on threat‐induced risk aversion differed by risk situation (risk‐with‐ambiguity and risk‐with‐no‐ambiguity), since those contexts have differing neurobehavioral impacts (Lauharatanahirun et al. 2025).

## 2 Method

### 2.1 Participants

Data were collected from 120 physically healthy adults who were either university students or non‐students from the surrounding community. Before enrollment, participants were randomly assigned to complete the study procedures during either a day session or night session. Day sessions could occur at any time between 8:30 AM and 4:30 PM, and night sessions could occur between 7:00 PM and 12:00 AM. Participants were recruited via an online recruitment platform (StudyFinder), social media, posted flyers, and word‐of‐mouth. Exclusionary criteria included: (1) a current and/or previous diagnosis of a cardiovascular, metabolic, or neurological condition, (2) use of nicotine products, and (3) diagnosis of COVID‐19 in the 2 weeks before the study. Prior to the lab visit, participants were required to abstain from: (1) alcohol for 24 h, (2) caffeine for 6 h, (3) eating for 2 h, and (4) vigorous exercise for 2 h. Exclusionary criteria were measured with an online prescreening questionnaire prior to enrollment. Participants confirmed abstention procedures (e.g., no caffeine for 6 h) by completing a brief questionnaire at the start of the lab visit. Participants who did not satisfy the abstention requirements were rescheduled to complete the study at a future date. Twenty‐three participants were excluded from the analysis due to completely missing or excessively noisy data arising from technical difficulties. That procedure left 97 participants (Day: *n* = 43, Night: *n* = 54). We also sought to ensure that the day and night sessions were temporally distinct to maximize internal validity; we therefore excluded participants whose sessions occurred during astronomical transition periods (e.g., sunset period). Specifically, day participants were only included in the analysis if they started study procedures after the end of civil dawn (i.e., end of morning civil twilight) AND ended procedures before the start of sunset. Night participants were only included if they started procedures after the end of civil dusk (i.e., end of evening civil twilight) and ended before the start of sunrise. The civil dawn/dusk end times provide practical thresholds for the start of daytime and nighttime according to societal, governmental, and research norms (https://www.ecfr.gov/current/title‐1) (Kimball 1916; Tan et al. 2013; Andre and Owens 2001). The clock times associated with these astronomical events (e.g., civil twilight end) were acquired from an online database provided by the U.S. Naval Observatory Astronomical Operations Department (https://aa.usno.navy.mil/data). Astronomical times were then compared to study procedure start/end times to identify sessions that did not satisfy our requirements. This procedure excluded ten participants in the night group who started procedures before civil dusk's end (a typical cutoff point for the start of nighttime)—leaving 87 participants in the final analysis (Day: *n* = 43, Night: *n* = 44).

### 2.2 Procedure

The study took place in a temperature‐controlled room with the lights on and “blackout” curtains drawn to prevent participants from seeing outside sunlight or lack thereof. The lights were kept on during study procedures for both the day and night conditions. These controls mitigated ambient lighting, or light versus dark, as a confound of time‐of‐day effects, which is our primary focus. Each session was overseen by two trained experimenters. If the participant satisfied the abstention criteria (see above), then they were attached to the physiological recording equipment. Abstention criteria were verified by having participants complete an electronic screening questionnaire. If they did not satisfy all criteria, then their laboratory session was rescheduled to a later date. They next completed self‐report questionnaires on a desktop computer while acclimating to the study setting. After questionnaires were completed, the experimenters instructed participants on the image‐decision task using a detailed slideshow, after which participants completed 3 practice trials and asked clarifying questions if they had any. Participants then completed the image‐decision task, which was segmented into 3 separate conditions that varied in the type of image presented: neutral, injury threat, infection threat. Each condition presented images from the same category (e.g., infection threat). Two‐minute resting baselines preceded each condition to mitigate fatigue effects. Participants wore noise canceling headphones during the entire task to prevent distraction due to extraneous noise. After completing the task, the experimenters removed the physiological equipment and compensated the participant with $25 in cash. Participants also received a bonus compensation of up to $5 in cash, which was calculated based on a randomly selected trial from the task (see task details below). Participants were then debriefed by the experimenters and exited the laboratory room. The study session lasted approximately 90 min.

### 2.3 Risk Assessment Task: Affective Images and Risky Decision‐Making

To study risk assessment, we used a novel computerized task that combined passive affective image viewing with economic lottery choices, with those choices being adapted from a single‐shot uni‐directional trust style game (Holt and Laury 2002; Holt 2019; Lauharatanahirun et al. 2012, 2025; Berg et al. 1995). See Figure 1. The task was scripted and implemented in MATLAB (version R2021b) on a 1920 × 1080p DELL PC monitor with a 60 Hz refresh rate. In each trial, participants were presented with an affective image followed by an economic decision. This formed two separate trial phases: an image viewing phase and a decisional phase. In the image viewing phase, varying image types (neutral, injury threat, infection threat; see Affective Stimuli section) were presented, with threat images putatively eliciting RA. Threat‐induced bradycardia was measured during the image viewing phase. The decisional phase can be considered a form of decisional priming where earlier threat images potentially modulate later choice, and these modulated choices estimated threat‐induced risk aversion. Participants were instructed that their goals were to attend to the images without averting the gaze, and to earn as much money possible such that each decision was independent from all other decisions.

In the decisional period of the trial, participants were asked to make decisions to either keep an initial endowment that ranged between $5 and $15 (safe option) or invest their allotted endowment in a gamble for the chance to earn more money (risky option). For each gamble, there was a high and low monetary payoff that were each associated with a probability. Probabilities of each potential outcome were represented using pie charts, where there were 10 slices in each pie that corresponded to a probability of 10% (see Figure 1). The “keep” pie was the safe option because it guaranteed the participant would retain the given monetary amount for that trial (e.g., 100% chance of earning $10). There was no uncertainty associated with the “keep” option. The “invest” pie was riskier because it was uncertain whether participants would earn less or more than their endowment on that trial (e.g., 60% of earning $14.70 and 40% of earning $2.60). To manipulate the risk situation, we changed whether the pie pieces (probability information) were occluded or not, such that half of the trials corresponded to each risk situation: no ambiguity versus ambiguity. Under risk‐with‐no ambiguity, none of the pie pieces were occluded. Under risk‐with‐ambiguity, 60% of the pie was occluded, and the occluded pieces of the pie could belong to high or low payoffs; the participants would not know which payoff wash hidden, thus increasing the level of uncertainty. In alignment with Induced Value Theory (Smith 1976), participants were told that they would receive an additional bonus payment of up to $5 based on their earnings from three randomly selected trials. This incentive structure allows participants' choices in the experiment to directly affect their monetary rewards, which promotes natural decision‐making akin to real‐world environments. Less focal to our core research question, participants were told in half of the trials (within image condition) that the “invest” option represented the likelihood of receiving amounts determined by a non‐social agent, specifically by a computer, which was designated visually as a roulette wheel. In the other half of trials, participants were told that outcomes were determined by a social agent, or a previous participant which was designated as a human face. Time‐of‐day and threat image effects did not differ between social and non‐social conditions, |*B*s| < 0.15, *p*s > 0.220. Therefore, all risk aversion results collapsed across the social and non‐social conditions while including a regressor to account for nuisance variance introduced by that manipulation (see Statistical Analysis).

#### 2.3.1 Trial Structure

At the start of each trial (Figure 1), an image was presented on the center of the screen for 5 s, constituting the image viewing period. The affective content varied depending on the image condition. After the image disappeared, a fixation cross was presented on the screen for a jittered inter‐stimulus interval drawn from a normal distribution of 0.5 s ± 0.2 s. Participants then completed the decisional period of the trial. In each trial, participants were first given a certain monetary endowment that ranged between ($5 and $15) that could either be kept (safe option) or invested in an uncertain gamble for the chance to potentially earn more money (risky option). These decision options were represented as two pie charts on the screen and shown to participants for 1 s. To prompt participants to make their selections, the message “Keep or Invest?” appeared at the top of the screen above the two options for a maximum of 3 s. Participants then indicated their decision on a keyboard using the “1” key corresponding to “keep” (safe option) and the “2” key corresponding to “invest” (risky option). In accord with prior studies (e.g., Lauharatanahirun et al. 2012), the safe and risky options were always presented on the left and right sides of the screen, and the keep and invest responses always corresponded to the left and right keys. This was done to reduce cognitive load and to ensure that choices were not confounded by cognitive errors or switching abilities. If participants failed to make a choice in the 3 s period, then they were presented with a screen containing the message: “TOO LATE! NO DECISION MADE!”, and the participant received no money during that trial. To separate events of the decisional period of the trial, a fixation cross was presented in the center of the screen for a jittered inter‐stimulus interval drawn from a normal distribution of 0.5 s ± 0.2 s. Once a decision was made, the trial ended, and the next trial commenced after a jittered inter‐trial interval drawn from a normal distribution with a mean of 1 s ± 4 s.

Participants completed a total of 180 trials across three conditions: injury threat (60 trials), infection threat (60 trials), and neutral (60 trials). Images of the same type were chunked together such that a single condition depicted only one image type (e.g., injury threat) across its 60 successive trials. The three conditions were separated by baseline periods. Serially presenting the same types of images back‐to‐back, as opposed to randomizing them on a trial‐to‐trial basis, served two purposes. It aimed to mitigate carryover effects between differing image types (e.g., threat onto neutral), and it facilitated the study of habituation to repeated images of the same type. Increasing the odds of habituation was critical given our hypotheses regarding weakened habituation of RA at night. The order of the image type conditions was randomized across participants. After each condition (*N* = 60 trials), participants received feedback about task earnings from a randomly selected trial in the preceding condition. The feedback was presented on a black screen with the text: “This is the end of block __ of 3. Your randomly selected payoff for this block is:__.” The randomly selected payoffs served as the bonus compensation, which could total to $5 in cash across conditions.

### 2.4 Affective Image Stimuli

The emotional images (*N* = 180) used in the task were color photographs acquired from Shutterstock, Dreamstime, and Pexels, online providers of high‐quality, royalty‐free images. Images were resized to a 1:1 aspect ratio (500 × 500 pixels). Injury threat images depicted stimuli that could inflict violent physical harm, including weapons, aggressive humans, and predators. Infection threat images depicted stimuli that could transmit infectious disease and cause illness. Neutral images involved mundane stimuli that have a lower probability of causing physical harm and infection, such as inanimate objects that appear in the household or outside, nature scenes, and human actors with emotionally neutral poses and/or facial expressions.

The 180 images used in this study were selected from a larger pool of images (*n* = 270) based on threat ratings of each image in a prior sample of participants (*n* = 734) (Spangler et al. 2024). The images were rated on negative emotions (fear, disgust) and threat appraisals (injury risk appraisal, illness risk appraisal) in this separate sample (Spangler et al. 2024). Ratings were made on Likert scales ranging from 0 to 100. For injury threat, we selected the images with the highest injury threat ratings, represented as the average score across a fear item (“The image made me feel scared.”) and an appraisal‐of injury‐risk item (“The scene and/or object in the image could hurt someone”). For infection threat, we selected the images with the highest infection threat ratings, represented as the average score across a disgust item (“The image made me feel disgusted”) and an appraisal‐of‐illness risk item (“The scene and/or object in the image could make someone sick”). For neutral, we selected the images with the lowest threat score, represented as the average score across all four items that were noted above.

We sought to validate the that the selected images activated the expected threat states. Multilevel regression models therefore tested differences between the image conditions in each of the four ratings (natural logarithm transformed to correct for skew); the analysis leveraged data from the prior sample (Spangler et al. 2024). The results indicate that, as expected, injury threat images had significantly higher fear and injury appraisal ratings compared to neutral images (fear: *B* = 1.97, *p* < 0.001; injury appraisal: *B* = 1.95, *p* < 0.001) and infection threat images (fear: *B* = 0.59, *p* < 0.001; injury appraisal: *B* = 0.82, *p* < 0.001). Also as expected, infection threat images had significantly higher disgust and infection appraisal ratings compared to neutral images (disgust: *B* = 2.35, *p* < 0.001; illness appraisal: *B* = 1.81, *p* < 0.001) and injury threat images (disgust: *B* = 0.77, *p* < 0.001; illness appraisal: *B* = 1.14, *p* < 0.001). This analysis suggests that the selected images in each condition are effective at eliciting negative emotions and threat appraisals tied to RA. They also showcase the separateness of injury and infection threat images in activating different threat states.

### 2.5 Physiological Recording

Electrocardiography (ECG) was continuously collected using a modified bipolar Lead II configuration. Lead wires were attached to adhesive spot electrodes below each clavicle and at the left ribcage. The signal was amplified (Gain = 2000) and filtered (0.05–150 Hz) with hardware (ECG 100C, BIOPAC systems Inc., Goleta, CA) before being digitized by the MP160 acquisition unit. The digitized signal was then routed to a laptop and recorded for offline processing using the AcqKnowledge Software (BIOPAC Systems Inc., Goleta, CA). In the offline processing, we applied a digital bandpass filter (0.5–35 Hz) to remove noise and then identified the R‐spikes using a modified Pan‐Tompkins algorithm. Successive R‐spikes were used to calculate the RR interval time courses for each trial. Artifact correction followed a careful two‐step procedure and generated corrected RR interval time courses. First, misclassified R‐spikes were manually corrected in AcqKnowledge by trained research assistants. Second, artifactual RR intervals were replaced with cublic spline interpolation; artifactual intervals were defined as intervals less than 300 ms, greater than 2000 ms, or more than 30% different from the last RR interval (Haaksma et al. 1995). Artifactual values constituted less than 1% of the ECG records. To further ensure validity of the cardiac data, image trials with more than three consecutive artifactual values were excluded from the bradycardia analyses; this procedure removed less than 1% of the trials. RR interval and not heart rate (HR) was used as our measure of cardiac chronotropy, because RR intervals have superior distributional properties, are more clearly and directly related to underlying vagus nerve traffic, and better reflect the physiological unit of cardiac chronotropy (i.e., heartbeats) (Berntson et al. 1995; Jennings et al. 1974). Although not of primary relevance to the current study, impedance cardiography was also collected using four adhesive spot electrodes on the posterior neck and back.

### 2.6 Measures

#### 2.6.1 Cardiac Response (“Bradycardia”) During Image Viewing

The corrected RR interval (milliseconds) time courses were used to compute cardiac deceleration, or bradycardia, scores. First, deceleration scores were computed by subtracting a pre‐image baseline from all RR intervals during the 5‐s image viewing period on a trial‐to‐trial basis. The pre‐image baseline was defined as the last complete RR interval before the image onset. This pre‐image baseline period occurred during the 1‐s inter‐trial interval (ITI) before each image, when participants passively viewed a black screen with a fixation cross. Second, we selected the maximum (i.e., peak) deceleration score that occurred during the image viewing period (5 s) for each image. When selecting the maximum score, we excluded the first deceleration score during the image since this likely reflects a “transient detecting response” and not attentional orienting that is germane to RA (Bradley 2009). The maximum deceleration score served as our index of “bradycardia”—how much RR interval lengthened in response to image viewing. These procedures were carried out for each image separately such that every trial had a bradycardia score.

#### 2.6.2 Decision‐Making (“Risk Aversion”) After Image Viewing

Decision‐making was measured as a binary variable for each trial, in terms of whether the participant chose the safe (coded as 0) or risky (coded as 1) option. The degree of risk aversion was operationalized as a lower likelihood (log‐odds) of choosing the risky option across trials, which we estimated with a logistic regression (described in the statistical analyses below).

#### 2.6.3 Self‐Report Questionnaires

Approximately 3.6% of item‐level survey responses were missing across the questionnaires. The anxiety subscale of the DASS‐21 was our primary focus in the survey data, and only 1.1% of this subscale's item responses were missing. To prevent a loss of statistical power and to minimize bias due to listwise deletion (Van Ginkel et al. 2020), missing values were filled in with multiple imputation using the multivariate imputation by chained equations method (Van Buuren 2007). The average value across five separate imputations served as the final imputed value for each missing score. Survey total scores (e.g., total DASS‐21 anxiety scores) and Cronbach's alphas both leveraged the imputed item‐level responses.

##### 2.6.3.1 Anxiety Symptoms

The anxiety subscale of the 21‐item version of the Depression, Anxiety, and Stress Scale (DASS‐21) has been used to index anxiety symptoms in various somatic and experiential domains (Antony et al. 1998; Lovibond 1995). Participants rated seven statements on the degree to which they applied to them over the past week, e.g., “I felt I was close to panic”; “I felt scared without any good reason.” Ratings were made on a four‐point Likert scale (0 to 3) with higher ratings indicating a stronger presence of symptoms. In accord with established criteria, ratings were summed and multiplied by 2 to yield a total anxiety score for each participant. The total scores exhibited acceptable internal consistency, *Cronbach's alpha* = 0.72. We used established criteria to digitize the total scores into a clinically meaningful categorical variable (Lovibond 1995). Specifically, participants with a total score of less than 8 were categorized as sub‐threshold, and individuals with a score of 8 or greater were categorized as above‐threshold. This established cutoff distinguished participants with *sub‐threshold* anxiety (*n* = 54) apart from those with *above‐threshold* anxiety (*n* = 33) in the current sample. In prior research, individuals in the above‐threshold group (score of 8 or greater) are more likely to have an anxiety disorder than are their below‐threshold (score lower than 8) counterparts (Lovibond 1995; Clover et al. 2022; Mitchell et al. 2008). It can therefore be inferred that individuals in the above‐threshold anxiety group likely have a heightened vulnerability for clinical anxiety; this points to the benefit of binarizing the variable as opposed to using continuous anxiety scores. The appropriateness of the binary classification (instead of the continuous scores) is also supported in our data since the continuous scores evidenced a non‐normal distribution where approximately half of the participants had relatively low scores (total score < 5).

##### 2.6.3.2 Circadian Preference

Circadian preference was self‐reported on the previously validated Morningness‐Eveningness Questionnaire (MEQ) (Horne and Ostberg 1976; Duffy et al. 2001; Bailey and Heitkemper 2001). The questionnaire contains 19 questions that are rated on a Likert scale. The items assess the time of the day when individuals are most comfortable/alert/etc. and when they would prefer to engage in certain activities, namely waking up and sleeping but also day‐to‐day behaviors like exercise. Item scores were summed to index a continuous circadian preference. Lower scores indicate a stronger evening preference while higher scores indicate a stronger morning preference. The scale's internal consistency in the current sample was good, *Cronbach's alpha* = 0.84.

##### 2.6.3.3 State Emotion

State emotion was self‐reported on the state form of the Positive–Negative Affect Schedule (PANAS). Participants rated 20 emotion adjectives based on how much they felt each emotion in that current moment. Ratings were made on a 5‐point Likert scale, ranging from 1 = “very slightly” to 5 = “extremely.” We were specifically interested in the adjective items “Alert” and “Active” because they addressed subjective fatigue as a confound of the observed time‐of‐day effects.

### 2.7 Statistical Analyses

Prior to running each statistical model, variables were screened for extreme outliers based on Tukey's 3*interquartile range (IQR) rule, and outliers were Winsorized to the fence (25th and 75th percentile ± 3*IQR). Outliers were detected only for < 1% of the trial‐level cardiac deceleration scores. Winsorization did not affect any other variables.

#### 2.7.1 Nocturnal Enhancement of RA Metrics Across the Sample

The hypotheses regarding time‐of‐day effects on RA metrics were tested with two‐level multilevel regression models that disentangle within‐ and between‐person variance (De Leeuw et al. 2008). A multilevel approach also permitted testing of cross‐level interactions between within‐person (e.g., image condition) and between‐person (e.g., time‐of‐day group) variables. Distinct models were conducted with bradycardia and risk aversion as separate outcome measures. Across all models, fixed regression effects tested our hypotheses and are reported as unstandardized regression coefficients (B). Each model included a random intercept of participant. Random slopes of level‐1 effects were also included when testing cross‐level interactions involving time‐of‐day group (level‐2) (Heisig and Schaeffer 2019). Significant interactions were probed with simple slope analysis. All fixed effects were tested against zero using Sattherwaithe degrees of freedom, two‐tailed *p*‐values, and an alpha of 0.05. Models were fit with full maximum likelihood using the *lme4* (Bates 2010) and *lmerTest* (Kuznetsova et al. 2017) packages in RStudio. Models were conducted for each outcome measure (bradycardia and risk aversion) using a three‐step approach, as is detailed below.

##### 2.7.1.1 Threat‐Induced Bradycardia

Bradycardia—the peak RR deceleration score relative to a pre‐image baseline—was entered as the outcome measure. The magnitude of threat‐induced bradycardia was represented as the extent to which there was greater bradycardia towards threat versus neutral images. Such threat‐related effects were modeled using two orthogonal dummy code regressors for injury and infection threat images separately: *Injury* vs. *Neutral* and *Infection* vs. *Neutral*, respectively. Neutral images served as the reference group for both injury and infection contrasts, with each contrast being tested simultaneously in the same model. A binary *Time‐of‐Day* (day = 0, night = 1) regressor modeled nocturnal effects as differences in bradycardia between the night and day groups. Another binary dummy regressor *Block* (first half of trials = 0, second half of trials = 1) modeled habituation as differences in bradycardia between earlier and later trials. Cross‐product interactions between these terms tested moderation effects. Specifically, the two‐way interactions between Injury/Infection contrasts and *Time‐of‐Day* modeled the nocturnal effects on average threat‐bradycardia for injury and infection threats separately. Three‐way interactions between Injury/Infection contrasts, *Block*, and *Time‐of‐Day* modeled nocturnal effects on the extent to which threat‐bradycardia habituated across blocks; as before, those interactions were tested separately for injury and infection threat images in the same model: *Injury* vs. Neutral*Block*Time‐of‐Day and *Infection* vs. Neutral*Block*Time‐of‐Day.

A three‐step approach generated separate multilevel models for threat‐induced bradycardia. That approach allowed us to comprehensively test all hypotheses with statistical clarity and rigor, while also providing a final model that is better specified and likely more accurate in accord to the data. First, we fit an *average model* that ignored habituation (without *Block* effects), which tested hypothesized time‐of‐day effects on *average* threat‐induced bradycardia/risk aversion. Here, the 2‐way interaction between image contrasts and *Time‐of‐Day* group (0 = day, 1 = night) tested day versus night differences in threat‐induced bradycardia averaged across blocks of each image condition. Second, we next fit a *maximal habituation model* to test cross‐level interactions representing time‐of‐day (level 2) effects on habituation in threat‐induced bradycardia (level 1). Those time‐of‐day differences in threat‐induced bradycardia's habituation were modeled as the 3‐way interactions between image contrasts, *Block*, and *Time‐of‐Day* group. Third, we fit a *refined model* that removed cross‐level interactions that were deemed non‐significant in the maximal or average model; we also removed their associated random slopes. Removing non‐significant interactions in this manner has been recommended for linear regression models (Engqvist 2005; Hayes 2009). Deleting null (*p* > 0.05) interaction terms (e.g., Block*Time‐of‐Day) allows researchers to accurately test lower‐order terms (e.g., Time‐of‐Day) as main effects, referring to average effects that pool across the moderator (e.g., Block). This is because when interactions are retained in the model, even if non‐significant, lower‐order terms are inherently tested as conditional effects that vary between levels of the moderator, not as main effects (Cohen et al. 2013). For example, removing non‐significant interactions (e.g., *Injury* vs. Neutral*Block) allowed for proper testing of the main effects of image type (*Injury* vs. *Neutral*) across levels of *Block*. Removing inappropriate cross‐level interactions and their associated random slopes, representing a backward elimination/selection procedure, is also a validated practice for improving statistical power and parsimony of multilevel models (Matuschek et al. 2017; Diggle 2002). We therefore report statistics from the refined models except when reporting non‐significant effects relating to an interaction term, which come from the average or maximal habituation model.

##### 2.7.1.2 Threat‐Induced Risk Aversion

Time‐of‐day effects on threat‐induced risky decision‐making were tested in separate multilevel logistic regression models. The log‐odds of risky choice (safe choice = 0, risky choice = 1) served as the outcome measure and indexed the degree of risk aversion. These logistic regression models used the same regressors as those testing bradycardia above, with the addition of the social versus non‐social contrast to account for nuisance variance introduced by that manipulation. The magnitude of threat‐induced risky decision‐making was indexed as the extent to which there was a lower likelihood of choosing the risky option following threat versus neutral images. Dummy code regressors modeled those effects separately for injury and infection images but did so simultaneously in each multilevel model. As with the bradycardia models above, we also leveraged a three‐step modeling approach where we focused on a final refined model.

##### 2.7.1.3 Sensitivity Analyses

Follow‐up sensitivity analyses were conducted to confirm that significant time‐of‐day effects from the multilevel models were true‐positive effects. There may be concern about overfitting the multilevel models with *N* < 100, especially when using maximum likelihood estimation. Our sensitivity analyses therefore took the form of simpler analyses of variance (ANOVA). In our view, if similar time‐of‐day results are obtained with the ANOVA, then the significant multilevel results are likely true‐positives. These ANOVAs used the same outcome and predictor variables as those used in the analogous multilevel models. Once exception is that risk aversion was represented as the proportion of trials in which the risky option was chosen (since ANOVA cannot handle binary outcomes, e.g., safe [0] vs. risky [1]).

#### 2.7.2 Nocturnal Enhancement of RA‐Anxiety Relationships

Inter‐person relationships first required computing average RA scores. Threat‐induced bradycardia, as an individual difference metric, was calculated by subtracting the mean bradycardia score for neutral images (average peak RR deceleration across neutral images) from the corresponding threat image score (e.g., average peak RR deceleration across infection threat images). The same approach calculated individual differences in threat‐induced changes in risky choice. These metrics were calculated separately for injury threat and infection threat images, and they characterized individual differences in RA physiology and RA behavior. Next, hypotheses for time‐of‐day effects on inter‐person associations between average RA measures and anxiety were tested with logistic regression models. The number of participants in each group by anxiety and time‐of‐day is as follows: *Day, Sub‐Threshold Anxiety*: *N* = 30. *Day, Above‐Threshold Anxiety*: *N* = 13. *Night, Sub‐Threshold Anxiety*: *N* = 24. *Night, Above‐Threshold Anxiety*: *N* = 20. Separate models were tested for each threat image type and RA measure. The log‐odds of belonging to the above‐threshold anxiety group (below‐threshold = 0, above‐threshold = 1) served as the dependent measure in each model. Interactions between time‐of‐day (day = 0, night = 1) and average bradycardia to threat images were modeled as cross‐product interactions. Significant interactions were probed with simple slope analysis. For example, a significant positive association between an RA measure, such as threat‐induced bradycardia, and anxiety group indicated that individuals with stronger bradycardia were more likely to be members of the above‐threshold anxiety group than those with weaker bradycardia. Two‐tailed *p*‐values and 95% CIs, with an alpha of 0.05, were used to test unstandardized regression coefficients against zero.

## 3 Results

Participant characteristics and self‐report variables are summarized in Table 1. None of the variables significantly differed between the day and night groups. We also tested PANAS items “alert” and “active” to address subjective fatigue as a confound of time‐of‐day effects. The day and night group did not significantly differ in feelings of being alert, *Welch t* = −0.60, *p* = 0.550, 95% CI [−0.60, 0.32], or in feelings of being active, *Welch t* = 1.50, *p* = 0.139, 95% CI [−0.12, 0.84].

**TABLE 1:** Descriptive statistics for participant characteristics.

	Combined (*N* = 87)			Day (*N* = 43)			Night (*N* = 44)			Day vs. night comparisons
	N	Mean (SD) or %	Median	N	Mean (SD) or %	Median	N	Mean (SD) or %	Median	*t*‐test or *χ* ^2^
Age		22.39 (5.57)	20		22.53 (4.84)	21		22.25 (6.25)	20	*t* = 0.24, *p* = 0.813
Gender										*χ* ^2^ = 0.09, *p* = 0.761
Female	60	69%		29	67%		31	70%		—
Male	27	31%		14	33%		13	30%		—
Racial identity										*χ* ^2^ = 1.25, *p* = 0.264
Caucasian/White	62	71%		33	77%		29	66%		—
African American/Black	7	8%		2	5%		5	11%		—
Asian	14	16%		6	14%		8	18%		—
Multi‐racial/other	4	5%		2	5%		2	5%		—
Education										*χ* ^2^ = 1.51, *p* = 0.218
High school or equivalency	60	69%		27	63%		33	75%		—
Associate's	2	2%		1	2%		1	2%		—
Bachelor's	8	9%		7	16%		1	2%		—
Master's	14	16%		7	16%		7	16%		—
Doctoral	2	2%		1	2%		1	2%		—
Professional (MD, JD, etc.)	1	1%		0	0%		1	2%		—
Income										*χ* ^2^ = 2.60, *p* = 0.107
Less than $5000	42	48%		17	40%		25	57%		—
$5000 through $11,999	22	25%		13	30%		9	20%		—
$12,000 through $15,999	3	3%		1	2%		2	5%		—
$16,000 through $24,999	6	7%		4	9%		2	5%		—
$25,000 through $34,999	8	9%		4	9%		4	9%		—
$35,000 through $49,999	2	2%		1	2%		1	2%		—
$50,000 through $74,999	4	5%		3	7%		1	2%		—
Anxiety score (DASS‐21)		6.23 (5.27)	4		5.58 (5.43)	4		6.86 (5.09)	6	*t* = −1.14, *p* = 0.259
Anxiety group										*χ* ^2^ = 2.14, *p* = 0.144
Sub‐Threshold	54	62%		30	70%		24	55%		—
Above‐Threshold	33	38%		13	30%		20	45%		—
Morningness‐Eveningness		46.82 (9.73)	47		47.56 (9.48)	47		46.09 (10.03)	47.5	*t* = 0.70, *p* = 0.485

*Note:* Differences in continuous variables between day and night groups were tested with two‐sample *t*‐tests (df = 85). Differences in categorical variables were compared with chi‐square tests of independence (*χ* ^2^) (df = 1). Before conducting chi‐square tests, categorical variables with more than two levels were transformed into binary variables because they had unbalanced counts across their levels. Those binary variables are as follows—Racial Identity: White (*N* = 62) versus not White (*N* = 25); Education: High school (*N* = 60) versus beyond high‐school (*N* = 27); Income: less than $5000 (*N* = 42) versus $5000 or above (*N* = 45).

### 3.1 Time‐Of‐Day (Day Versus Night) Effects on Threat‐Induced Bradycardia

**FIGURE 2:**
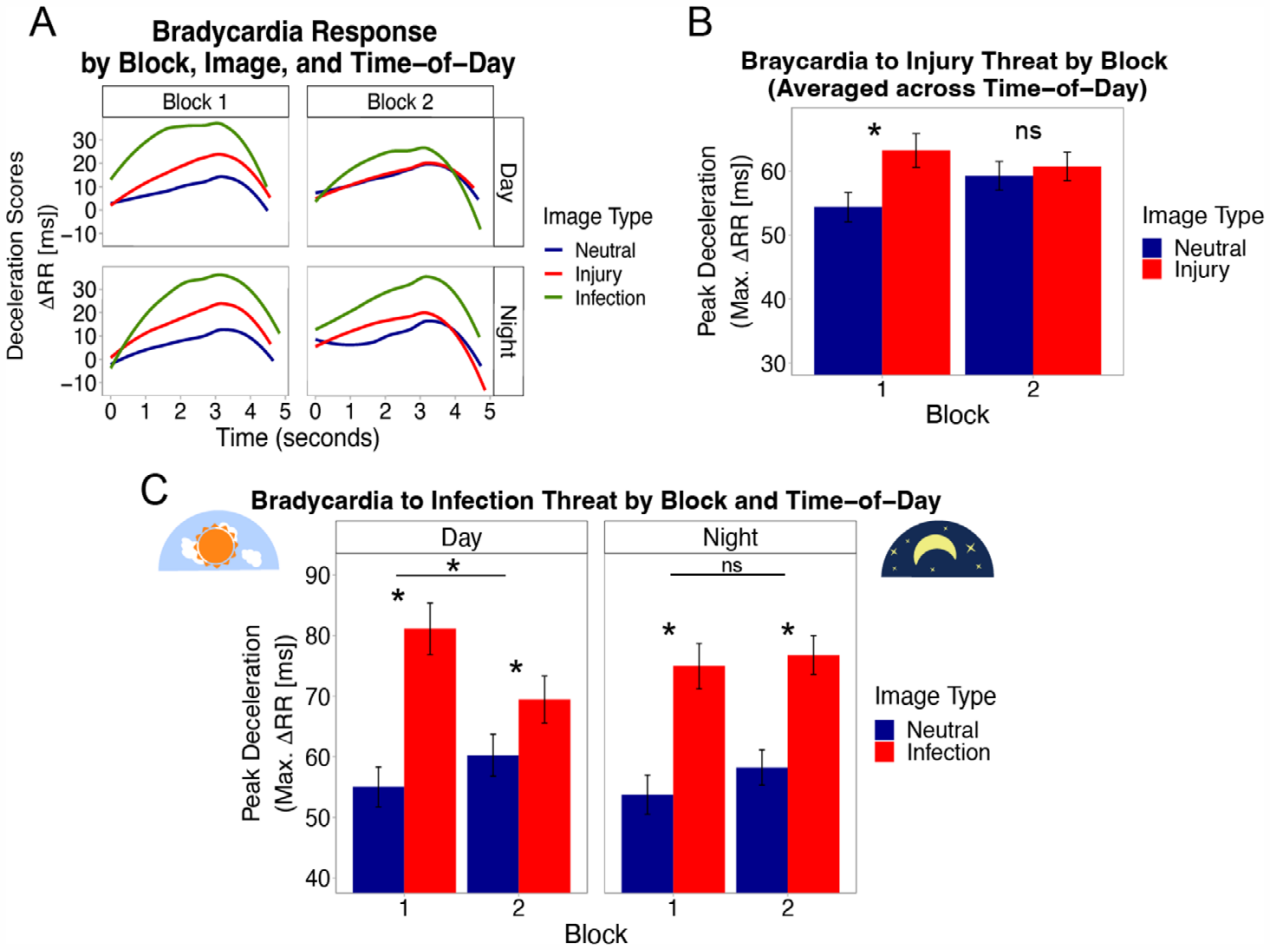
**Threat‐induced bradycardia.** **Panel (A)**: Bradycardia time courses during image viewing. Lines reflect mean time courses in raw RR deceleration scores across participants and images, estimated as smoothed average scores using a LOESS procedure. **Panel (B)**: Injury threat bradycardia habituates between blocks, collapsing across time‐of‐day. The bars depict mean bradycardia by image type and blocks, calculated as the average of the peak RR deceleration scores, collapsing across images, time‐of‐day, and participants. The whiskers depict within‐person standard errors. Below, conditional effects probe the *Injury* vs. Neutral*Block interaction. **Panel (C)**: Infection threat bradycardia habituates during the day but not at night. Bars depict mean bradycardia by image type, blocks, and time‐of‐day; here, mean levels of bradycardia reflect average peak RR deceleration scores collapsing across images and participants. The whiskers depict within‐person standard errors. *p < 0.05 (two‐tailed).

Table 2 summarizes the refined model that removes non‐significant interactions. That model reflects our core results because they arise from the most parsimonious model of the three. All significant results reported below hence come from the refined model; non‐significant effects reported below come from the average and maximal habituation models (see Tables S1 and S2). For completeness, we also plotted bradycardia results using heart rate (Figure S2). Those plots demonstrate similar patterns as our focal results in Figure 2, which use RR intervals.

**TABLE 2:** Refined Multilevel Model: Threat‐Induced Bradycardia.

Dependent measure: Bradycardia (peak cardiac deceleration during image)						
Fixed effects	*B*	SE	*95% CI: Low*	*95% CI: High*	df	*p*
Intercept	50.26	4.56	41.32	59.20	97.45	< 0.001*
Injury vs. Neutral	8.86	2.14	4.66	13.06	15275.25	< 0.001*
Infection vs. Neutral	27.41	5.43	16.77	38.04	94.20	< 0.001*
Block	6.39	2.97	0.56	12.21	159.16	0.033*
Injury vs. Neutral*Block	−7.40	3.03	−13.34	−1.45	15276.82	0.015*
Infection vs. Neutral*Block	−18.04	4.62	−27.09	−8.99	109.36	< 0.001*
Time‐of‐Day (ToD)	8.11	6.24	−4.11	20.33	87.00	0.197
ToD*Block	−3.10	3.60	−10.15	3.95	87.14	0.391
Infection vs. Neutral*ToD	−7.45	7.48	−22.11	7.20	87.01	0.322
Infection vs. Neutral*Block*ToD	16.51	6.13	4.48	28.53	87.13	0.009*
						
	Variance				Value	
Participant (intercept)	745.54			Deviance	180680.7	
Infection vs. Neutral (slope)	916.70			AIC	180722.7	
Block (slope)	81.13			ICC	0.10	
Infection vs. Neutral* Block (slope)	217.59					

*Note:* A multilevel regression model was fit with maximum likelihood estimation. Unstandardized regression coefficients (*B*) are provided. Dependent Measure: Bradycardia refers to the peak cardiac deceleration (maximum RR interval change score) during the image relative to a pre‐image baseline.

Abbreviation: ToD, time‐of‐day group.

* *p* < 0.05 (two‐tailed).

#### 3.1.1 Time‐of‐Day Does Not Modulate Injury Threat‐Induced Bradycardia

Averaging across blocks, the day and night groups did not significantly vary from one another in how much bradycardia differed between injury threat and neutral images, *Injury* vs. Neutral*Time‐of‐Day: B = 2.78, *p* = 0.599, 95% CI [−7.52, 13.07]. Similarly, the day and night groups did not differ in how much these bradycardia differences (between injury threat versus neutral images) changed between blocks 1 and 2, *Injury* vs. Neutral*Block*Time‐of‐Day: *B =* −4.78, *p* = 0.540, 95% CI [−20.01, 10.45]. See Figure 2A. These results indicate that the day and night groups did not differ in average bradycardia to injury threat or in the extent to which bradycardia to injury threat habituated between blocks. Since there were no time‐of‐day effects, we pooled across the day and night groups by removing the time‐of‐day interaction in the refined model (Table 2). After doing so, we found that injury threat‐induced bradycardia habituated between blocks, *Injury* vs. Neutral*Block: *B =* −7.40, *p =* 0.015, 95% CI [−13.34, −1.45]. Probing this interaction revealed that there was significantly stronger bradycardia to injury threat versus neutral images at block 1, *Injury* vs. *Neutral*: *B* = 8.86, *p* < 0.001, 95% CI [4.66, 13.06], and this effect weakened (i.e., habituated) to become non‐significant at block 2, *Injury* vs. *Neutral*: *B* = 1.47, *p* = 0.495, 95% CI [−2.74, 5.67]. Figure 2B depicts that habituation effect, which averages across the day and night groups.

#### 3.1.2 Habituation in Infection Threat‐Induced Bradycardia is Weakened at Night but Not During the Day

Averaging across blocks, the day and night groups did not significantly vary from one another in how much bradycardia differed between infection threat versus neutral images, *Infection* vs. Neutral*Time‐of‐Day: B = 2.19, *p* = 0.741, 95% CI [−10.77, 15.16] (Figure 2A). That result suggests that time‐of‐day did not modulate average infection threat‐induced bradycardia. However, additional analyses revealed that, for individuals with elevated anxiety symptoms, the magnitude of average bradycardia to infection threat images is significantly larger at night. This effect appears in the section below entitled “Inter‐person associations between nocturnal RA and anxiety symptoms.”

We next turn to habituation in the infection threat effects. The day and night groups significantly differed in the degree to which bradycardia differences (to infection versus neutral images) changed between blocks, *Infection* vs. Neutral*Block*Time‐of‐Day: B = 16.51, *p* = 0.009, 95% CI [4.48, 28.53]. That interaction was significant in both the maximal habituation model (Table S2) and in the refined model (Table 2), but the latter interaction and the following conditional effects come from the refined model. See Figure 2C for these differential habituation effects by time‐of‐day, represented by the conditional 2‐way interactions (*Infection* vs. Neutral*Block) by time‐of‐day as well as lower‐order *Infection* vs. *Neutral* contrasts by time‐of‐day and block. Those conditional effects were tested as simple slopes and are detailed here. In the day group, there was a statistically significant *Infection* vs. Neutral*Block interaction, *B* = −18.04, *p* < 0.001, 95% CI [−27.09, −8.99]. This interaction was further probed with simple slopes in each block separately. In the day group, infection threat images evoked significantly stronger bradycardia than did neutral images at block 1, *Infection* vs. *Neutral*: *B* = 27.41, *p* < 0.001, 95% CI [16.78, 38.04], and that effect habituated (was significantly weakened in size) at block 2, *Infection* vs. *Neutral*: *B* = 9.37, *p* = 0.047, 95% CI [0.25, 18.50]. In the night group, the *Infection* vs. Neutral*Block interaction was not statistically significant, *B* = −1.53, *p* = 0.738, 95% CI [−10.50, 7.43], suggesting that the infection threat effects on bradycardia did not differ or habituate between the blocks. For completeness, we also report the simple slopes by block in the night group. There was significant bradycardia evoked by infection threat versus neutral images at block 1, *Infection* vs. *Neutral*: *B* = 19.96, *p* < 0.001, 95% CI [9.44, 30.47], and this effect remained significant and comparable in size at block 2, *Infection* vs. *Neutral*: *B* = 18.42, *p* < 0.001, 95% CI [9.39, 27.45]. These results indicate that the night period—unlike the day period—lacked the typical pattern of habituation involving decreases in threat‐induced bradycardia across repeated infection threat images.

To confirm the time‐of‐day effect, a sensitivity analysis was conducted for the three‐way interaction in the multilevel model, *Infection* vs. Neutral*Block*Time‐of‐Day. We ran a simpler three‐way mixed ANOVA with a 2 (*Image Condition*: Infection Threat, Neutral) × 2 (*Block*: 1, 2) × 2 (*Time‐of‐Day*: Day, Night) structure. The ANOVA demonstrated a significant three‐way interaction between Time‐of‐Day, Image Condition, and Block, *F*(1, 85) = 3.97, *p* = 0.0497, *η* ^*2*^_*G*_ = 0.002. We therefore probed the interaction with 2‐way ANOVAs in the day and night groups separately. There was a statistically significant interaction between *Image Condition* and *Block* in the day group, *F*(1, 42) = 14.26, *p* = 0.001, *η* ^*2*^_*G*_ = 0.01, but not in the night group, *F*(1, 43) = 0.23, *p* = 0.634, *η* ^*2*^_*G*_ < 0.001. Those results suggest infection threat (versus neutral) effects on bradycardia significantly differed between blocks in the day but not the night group. In the day, follow‐up *t*‐tests reveal that this variation between blocks reflected habituation: bradycardia was significantly larger to infection threat versus neutral images at block 1, *t*(42) = −4.34, *p* < 0.001, 95% CI [−38.24, −13.97], *Cohen's d* = −0.66, whereas that neutral‐versus‐threat difference decreased to become non‐significant at block 2, *t*(42) = −1.64, *p* = 0.108, 95% CI [−20.49, 2.09], *Cohen's d* = −0.25. At night, bradycardia was significantly larger to infection threat versus neutral images at block 1, *t*(43) = −4.15, *p* < 0.001, 95% CI [−31.53, −10.89], *Cohen's d* = −0.62, and this difference remained significant and comparable in size at block 2, *t*(43) = −3.96, *p* < 0.001, 95% CI [−28.00, −9.09], *Cohen's d* = −0.60. Overall, the ANOVA results support the multilevel model statistics and suggest weakened habituation of infection threat bradycardia at night versus day.

### 3.2 Time‐Of‐Day Effects on Threat‐Induced Risk Aversion

Table 3 summarizes the refined testing risk aversion as a dependent measure. As with the bradycardia results, all significant results reported below come from the refined model; otherwise, non‐significant effects reported below come from the average and maximal habituation models (Tables S3 and S4).

**TABLE 3:** Refined Multilevel Model: Threat‐Induced Risk Aversion.

Dependent measure: Risky choice (log‐odds of risky choice)					
Fixed effects	*B*	SE	*95% CI: Low*	*95% CI: High*	*p*
Intercept	−0.07	0.15	−0.35	0.22	0.636
Injury vs. Neutral	−0.19	0.08	−0.34	−0.04	0.014*
Infection vs. Neutral	−0.13	0.04	−0.21	−0.05	0.002*
Block	−0.08	0.03	−0.15	−0.01	0.021*
Time‐of‐Day (ToD)	−0.29	0.20	−0.68	0.11	0.153
Injury vs. Neutral*ToD	0.25	0.10	0.05	0.45	0.016*
**Random effects**				**Model fit**	
	Variance				Value
Participant (intercept)	0.84			Deviance	19637.3
Injury vs. Neutral (slope)	0.11			AIC	19655.3

*Note:* A logistic multilevel regression model was fit with maximum likelihood estimation. Unstandardized regression coefficients (*B*) are provided. Dependent Measure: The log‐odds ratio of risky choice (i.e., degree of risk aversion).

Abbreviation: ToD, time‐of‐day group.

* *p* < 0.05 (two‐tailed).

#### 3.2.1 Injury Threat‐Induced Decreases in Risky Choice Emerge During the Day but Not at Night

**FIGURE 3:**
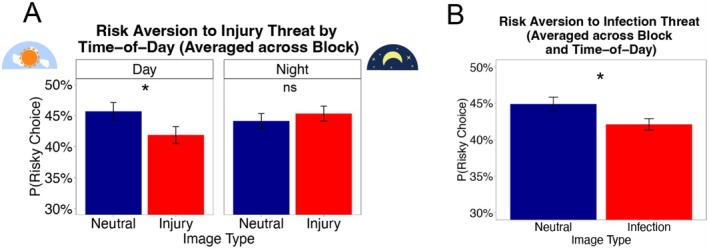
**Threat‐induced risk aversion**. **Panel (A)**: Average injury threat‐induced risk aversion (pooled across blocks) is apparent during the day but not at night. The bars depict the average probability of choosing the risk option by image type and time‐of‐day group, calculated as the mean percentage of risky decisions across images, blocks, and participants. The whiskers depict within‐person standard errors. Below, conditional effects probe the *Injury* vs. Neutral*Time‐of‐Day interaction. **Panel (B)**: There is evidence of infection threat‐induced risk aversion, pooling across blocks and time‐of‐day. The bars depict the average probability of choosing the risky option by image type, calculated as the mean percentage of risky decisions across images, blocks, time‐of‐day, and participants. The whiskers depict within‐person standard errors. *p < 0.05 (two‐tailed).

Averaged across blocks, the day and night groups significantly differed in how much the odds of risky choice differed between injury threat and neutral images, *Injury* vs. Neutral*Time‐of‐Day: *B* = 0.25, *p* = 0.016, 95% CI [0.05, 0.45]. However, the day and night groups did not significantly differ in how much this difference (*Injury* vs. *Neutral*) habituated between blocks, *Injury* vs. Neutral*Block*Time‐of‐Day: *B* = −0.22, *p* = 0.187, 95% CI [−0.55, 0.11]. We now focus on the former interaction effect that averaged across blocks, which is displayed and probed with conditional effects in Figure 3A. In the day group, viewing injury threat versus neutral images was related to a reduced likelihood of choosing the risky option, i.e., a pattern of threat‐induced decreases in risky decision‐making, *Injury* vs. *Neutral*: *B* = −0.19, *p* = 0.014, 95% CI [−0.34, −0.04]. In the night group, injury threat images did not influence risky choice, *Injury* vs. *Neutral*: *B* = 0.06, *p* = 0.417, 95% CI [−0.09, 0.21]—participants were just as likely to choose the risky option after viewing injury threat versus neutral images. These findings indicate that threat‐induced risk aversion was present during the day but not during the night period.

We sought to determine whether the latter effect was present under both risk situations, *risk‐with‐no‐ambiguity* and *risk‐with‐ambiguity* (Koch et al. 2017), which was disentangled with our task design. This exploratory analysis informs whether the time‐of‐day more strongly impacts aversion towards a certain type of risk. Multilevel models with the same structure as the refined model (Table 3) were conducted separately on each set of trials, those involving *risk‐with‐ambiguity* versus those involving *risk‐with‐no‐ambiguity*. For *risk‐with‐no‐ambiguity* trials, there was a significant *Injury* vs. Neutral*Time‐of‐Day interaction, *B* = 0.39, *p* = 0.005, 95% CI [0.12, 0.66]. The analogous interaction for *risk‐with‐ambiguity* trials was not statistically significant, *B* = 0.12, *p* = 0.379, 95% CI [−0.15, 0.39]. We next probed the significant interaction for *risk‐with‐no‐ambiguity trials* using simple slopes. In the day group, injury threat images were associated with a lower odds of risky choice than neutral images, *Injury* vs. *Neutral: B* = −0.23, *p* = 0.021, 95% CI [−0.43, −0.03]. In the night group, injury threat and neutral images were associated with the same odds of risky choice, *Injury* vs. *Neutral: B* = 0.15, *p* = 0.131, 95% CI [−0.05, 0.35]. Those results suggest that the time‐of‐day effect on threat‐induced risk aversion in Figure 3A is driven by risk trials where potential outcomes are known.

To confirm the time‐of‐day effect on risky choice, a sensitivity analysis was conducted for the two‐way interaction in the multilevel model, *Injury* vs. Neutral*Time‐of‐Day. Specifically, we ran a simpler two‐way mixed ANOVA with a 2 (*Image Condition*: Infection Threat, Neutral) × 2 (*Time‐of‐Day*: Day, Night) structure. The outcome measure focused on the proportion of risky choices during the *risk‐with‐no‐ambiguity* trials, since the multilevel effect above was significant only for these trials. The ANOVA demonstrated a significant two‐way interaction between Time‐of‐Day and Image Condition, *F*(1, 85) = 4.98, *p* = 0.028, *η* ^*2*^_*G*_ = 0.01, suggesting that injury threat effects differed between day and night conditions. Follow‐up *t*‐tests indicate that there was larger risk aversion following injury threat (versus neutral) in the day, *t*(42) = 1.71, *p* = 0.094, 95% CI [−0.01, 0.11], *Cohen's d* = 0.24, although this effect was not statistically significant. The analogous effect (injury versus neutral) was not significant in the night condition, *t*(43) = −1.43, *p* = 0.161, 95% CI [−0.07, 0.01], *Cohen's d* = −0.15. The ANOVA results are generally aligned with the multilevel model results in that they both demonstrate an Image Type*Time‐of‐Day interaction; however, the follow‐up *t*‐tests were not statistically significant. Taken together, the results mostly confirm stronger injury threat‐induced risk aversion during the day relative to the night, although this effect should be interpreted cautiously. They also underscore the heightened sensitivity of the multilevel model relative to the ANOVA approach.

#### 3.2.2 Time‐of‐Day Does Not Modulate Infection Threat‐Induced Risk Aversion

When averaging across blocks, there was no significant time‐of‐day effect on the difference in risky choice between infection threat versus neutral images, *Infection* vs. Neutral*Time‐of‐Day: *B* = −0.02, *p* = 0.835, 95% CI [−0.24, 0.19]. There was also no significant influence of time‐of‐day group on the degree to which the infection threat effect differed between blocks, *Injury* vs. Neutral*Block*Time‐of‐Day: *B* = −0.03, *p* = 0.856, 95% CI [−0.36, 0.30]. In other terms, time‐of‐day did not impact the degree of habituation in infection threat risk‐induced aversion. Pooling across day/night groups and blocks, participants were less likely to choose the risky option following infection threat versus neutral images, *Infection* vs. *Neutral*: *B* = −0.13, *p* = 0.002, 95% CI [−0.21, −0.05]. This result suggests that, across trials and time‐of‐day groups, infection threat images decreased risky choice relative to neutral images (Figure 3B). To clarify this effect, we re‐ran the model for risk‐with‐no‐ambiguity and risk‐with‐ambiguity trials separately. The *Infection* vs. *Neutral* effect was statistically significant for risk‐with‐ambiguity, *B* = −0.19, *p* = 0.002, 95% CI [−0.31, −0.07], but not for risk‐with‐no‐ambiguity, *B* = −0.08, *p* = 0.166, 95% CI [−0.20, 0.03]. In other words, the effect of infection threat on risky choice was specific to risk‐with‐ambiguity trials—when information about the outcome likelihoods was partially unknown.

### 3.3 Inter‐Person Associations Between Nocturnal RA and Anxiety Symptoms

Table 4 summarizes the logistic regression models that tested the relations between RA metrics (*Bradycardia*, *Risky Choice*) and the odds of anxiety group membership (0 = sub‐threshold, 1 = above‐threshold) as a function of time‐of‐day group. Separate models were conducted for bradycardia and risky choice towards injury threat versus infection threat. The models that pooled across time of days groups are summarized in the Supporting Information (Table S5).

**TABLE 4:** Logistic regression models: Associations between threat metrics and anxiety symptoms by time‐of‐day.

	Bradycardia models									
	Injury threat (model fit: AIC = 118.47)					Infection threat (model fit: AIC = 111.89)				
	*B*	SE	*95% CI: Low*	*95% CI: High*	*p*	*B*	SE	*95% CI: Low*	*95% CI: High*	*p*
Intercept	−0.85	0.34	−1.57	−0.20	0.013*	−0.86	0.34	−1.57	−0.22	0.011*
Threat‐induced bradycardia	0.02	0.01	−0.01	0.05	0.124	−0.01	0.01	−0.03	0.01	0.390
Time‐of‐day (0 = day, 1 = night)	0.66	0.46	−0.23	1.58	0.153	0.60	0.48	−0.34	1.55	0.212
Bradycardia*Time‐of‐day	−0.01	0.02	−0.05	0.02	0.479	0.05	0.02	0.01	0.08	0.008*

	Risk aversion models									
	Injury threat (model fit: AIC = 120.82)					Infection threat (model fit: AIC = 121.10)				
	*B*	SE	*95% CI: Low*	*95% CI: High*	*p*	*B*	SE	*95% CI: Low*	*95% CI: High*	*p*
Intercept	−0.88	0.34	−1.60	−0.24	0.010*	−0.84	0.33	−1.53	−0.21	0.012*
Threat‐induced risk aversion	−1.56	2.41	−6.61	3.32	0.519	−1.35	2.82	−7.03	4.26	0.631
Time‐of‐day (0 = day, 1 = night)	0.72	0.46	−0.17	1.65	0.119	0.66	0.45	−0.22	1.56	0.145
Risk aversion*Time‐of‐day	0.73	3.59	−6.44	7.89	0.839	1.19	4.03	−6.81	9.18	0.767

*Note:* All logistic regression models are conducted on the full sample (*N* = 87). In each model, the dependent measure is the log‐odds of membership in the above‐threshold anxiety group, signaling heightened risk for clinical anxiety.

* *p* < 0.05 (two‐tailed).

#### 3.3.1 The Nocturnal Setting Amplifies the Inter‐Person Association Between Infection Threat‐Induced Bradycardia and Anxiety

The relationship between bradycardia to injury threat and the odds of anxiety group membership did not differ between the day and night groups, as indicated by the non‐significant Bradycardia*Time‐of‐Day interaction, *B* = −0.01, *p* = 0.479, 95% CI [−0.05, 0.02]. We therefore re‐ran the model without the interaction to examine the bradycardia‐anxiety relationship pooling across day and night groups. Pooling across day/night groups, there was no significant association between bradycardia to injury threat and anxiety group, *Bradycardia*: *B* = 0.01, *p* = 0.116, 95% CI [−0.003, 0.03].

**FIGURE 4:**
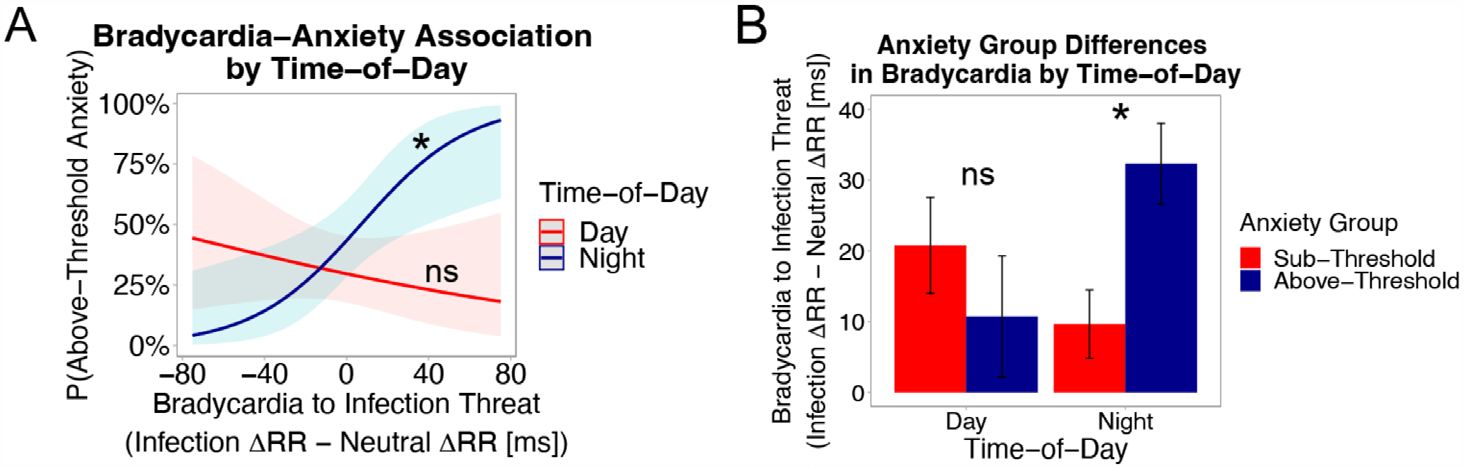
**Inter‐person associations between infection threat bradycardia and anxiety symptoms by time‐of‐day.** *Bradycardia to Infection Threat* = Average peak RR deceleration to infection threat subtracting out average peak RR deceleration to neutral images. *Anxiety Group* = whether individuals have anxiety symptoms that are sub‐threshold (0) or above‐threshold (1). *Time‐of‐Day* = whether threat bradycardia was measured during the day (0) or night (1). **Panel (A)**: Infection threat‐induced bradycardia during the night (but not during the day) is related to anxiety symptoms. The graph and statistics below reflect the conditional effects for the Bradycardia*Time‐of‐Day interaction. **Panel (B)**: Relative to their below‐threshold counterparts, individuals with above‐threshold anxiety have higher infection threat bradycardia at night but not during the day. Bars reflect average bradycardia scores by time‐of‐day and anxiety group collapsing across image of the same type, blocks, and participants. Whiskers indicate standard errors. *p < 0.05 (two‐tailed).

We next turn to the models testing bradycardia to infection threat and anxiety group. Infection threat‐induced bradycardia was more strongly related to anxiety group in the night versus day group, Bradycardia*Time‐of‐Day: *B* = 0.05, *p* = 0.008, 95% CI [0.01, 0.08]. Figure 4A plots the interaction's conditional effects (simple slopes) in each time‐of‐day group; they are also detailed here. In the night group, individuals with stronger bradycardia to infection threat images were more likely to be members of the above‐threshold anxiety group than those with weaker bradycardia to the same images, *Bradycardia*: *B* = 0.04, *p* = 0.009, 95% CI [0.01, 0.07]. In the day group, individuals with stronger bradycardia to infection images were not more (or less) likely to be in the above‐threshold anxiety group than were those with weaker bradycardia to the same images, *Bradycardia B* = −0.01, *p* = 0.390, 95% CI [−0.03, 0.01].

This significant interaction between infection threat bradycardia and time‐of‐day in predicting anxiety group was probed in two additional ways. First, we compared average levels of threat‐induced bradycardia between anxiety groups for day and night separately (Figure 4B). In the day group, there was no significant mean difference in infection threat‐induced bradycardia between the anxiety groups, *Welch t* = 0.92, *p* = 0.366, 95% CI [−12.36, 10.71]. In the night group, the mean difference in the same bradycardia scores between anxiety groups was statistically significant, *Welch t* = −3.04, *p* = 0.004, 95% CI [−37.81, −7.60]. Those results indicate that only in the night group did above‐threshold anxiety individuals have significantly elevated threat‐induced bradycardia compared to their below‐threshold counterparts. Thus, infection threat‐induced bradycardia better differentiated above‐ versus below‐threshold anxiety symptoms when bradycardia was assessed at night. Second, we compared average levels of infection threat‐induced bradycardia between day and night groups in the below‐ and above‐threshold groups separately. In the below‐threshold anxiety group, there was no significant difference in average infection threat‐induced bradycardia between day and night groups, *Welch t* = 1.34, *p* = 0.188, 95% CI [−5.61, 27.85]. In the above‐threshold anxiety group, averaged infection threat bradycardia was significantly elevated at night versus day, *Welch t* = −2.10, *p* = 0.047, 95% CI [−42.97, −0.31].

##### 3.3.1.1 Sensitivity Analysis

We sought to validate the significant time‐of‐day effect on the infection threat‐induced bradycardia and anxiety group. That is, we wanted to ensure that this effect persisted across both blocks (1 and 2) in each image condition. To that end, we reran the same logistic regression model in each block separately. There were significant Bradycardia*Time‐of‐Day interactions both at block 1, *B* = 0.03, *p* = 0.023, 95% CI [0.01, 0.06], and block 2, *B* = 0.03, *p* = 0.030, 95% CI [0.01, 0.07]. Simple slopes in each block revealed that individuals with higher bradycardia to infection threat were more likely to be in the above‐threshold anxiety group when bradycardia was assessed at night, Block 1: *B* = 0.02, *p* = 0.028, 95% CI [0.004, 0.05]; Block 2: *B* = 0.03, *p* = 0.028, 95% CI [0.006, 0.06]. When bradycardia was assessed during the day, this relation was not significant at either block 1, *B* = −0.01, *p* = 0.358, 95% CI [−0.03, 0.01], or block 2, *B* = −0.01, *p* = 0.509, 95% CI [−0.03, 0.01]. Together, these findings suggest that the night period enhanced the relationship between infection threat bradycardia and anxiety symptoms, irrespective of whether bradycardia was measured at earlier or at later trials in the image condition.

#### 3.3.2 Time‐of‐Day Does Not Modulate the Associations Between Threat‐Induced Risk Aversion and Anxiety

Across injury and infection threat measures, there were no day‐versus‐night differences in any of the relations between threat‐induced changes in risky choice and anxiety group, Risky Choice*Time‐of‐Day interactions: *B*s < 1.20, *p*s > 0.05 (Table 4). We next examined relationships collapsing across day/night groups. Injury threat‐induced change in risky choice was not significantly associated with the log‐odds of anxiety group membership, *Risky Choice: B* = −0.65, *p* = 0.706, 95% CI [−4.12, 2.79]. Similarly collapsing across day/night groups, infection threat‐induced change in risky choice was not significantly related to anxiety group, *B* = −0.79, *p* = 0.691, 95% CI [−4.78, 3.12].

## 4 Discussion

Risk assessment (RA) is the detection/analysis of ambiguous threat that guides optimal defense and represents a likely phenotype for anxiety disorders (Blanchard 2017; Blanchard and Meyza 2019). Human RA should be strengthened at night to protect against threats cloaked in darkness (Darwin 1871; Herrmann 2015; Nasar and Fisher 1993; Grillon et al. 1997). The current study tested this intuitive notion of “nocturnal enhancement” by inducing RA with threatening images during distinct day and night conditions. We captured RA's separate cognitive and behavioral aspects by examining how cardiac activity (threat‐induced bradycardia) and risky decision‐making (threat‐induced risk aversion) changed in response to threat images of varying types, injury threat and infection threat. We also asked whether the nighttime activates an RA phenotype, where inter‐person associations between RA metrics and anxiety vulnerability, measured with the DASS‐21, would be higher at night versus day. Our core hypothesis was that nocturnal enhancement of RA metrics (involving heightened average RA and weakened habituation of RA) and their association with anxiety would emerge across both threat types: injury and infection threat. However, we also explored whether such associations differed between threat types and whether time‐of‐day effects on risk aversion differed between risk situations that are important in the neuroeconomic literature: risk‐with‐no‐ambiguity versus risk‐with‐ambiguity.

Overall, we show that nocturnal enhancement of human RA is constrained to a particular RA component and threat type; our hypothesis of nocturnal enhancement was therefore only partially supported. The nocturnal period only enhanced the cardiac response to infection stimuli (suggesting attention to ambiguous threat) (Bradley 2009; Gladwin et al. 2016) and the degree to which this cardiac response was related to anxiety symptoms. Conversely, the nocturnal period did not strengthen the cardiac response to injury threat or its link to anxiety, likely because injury threats are less ambiguous and require less interpretation/attention. Evidence of nocturnal enhancement was absent for decision‐making variables. The night period did not strengthen risk‐averse decision‐making following threat, an aspect of RA reflecting behavioral caution (Holt and Laury 2014; Clark et al. 2012). Our findings therefore suggest that the nighttime enhances attentional orienting to ambiguous threats with little to no effect on cautious behavior. This study also suggests that the nocturnal period might activate a phenotype for anxiety that has implications for clinical diagnosis and early intervention. That is, individuals with heightened risk for anxiety disorders may demonstrate elevated attentional orienting to ambiguous threat at night but not during the day. A more detailed discussion of the findings appears below.

Consistent with prior research, we found evidence of threat‐induced bradycardia to both image types that generally habituated across time (Lang et al. 1997; Bradley 2009; Vila et al. 2007). We found several effects consistent with the hypothesized notion that the nocturnal period enhances the attentional aspect of RA vis‐à‐vis threat‐induced bradycardia. First, the nocturnal period weakened habituation in infection threat bradycardia. Habituation in threat responses, although normative, can be adaptively suppressed to sustain defensive reactions when continual defense is required for survival (if threats are unpredictable or intense) (De Boer et al. 1989; Deecke et al. 2002; Bertels et al. 2012). Our finding of weakened habituation in bradycardia at night might suggest an evolutionarily adaptive pattern of prolonged vigilance that prevents one from missing ambiguous threats in the visual environment (Bell et al. 2009; Dalmaijer et al. 2021). Second, higher average infection‐threat induced bradycardia (average across trials) was only detected for individuals with above‐threshold anxiety. Thus, across anxiety levels, the night may enhance attention by prolonging the duration but not the intensity level of attention towards infection threats. For high‐anxiety individuals specifically, however, the nighttime may heighten the overall intensity of attention towards infection threats. Importantly, similar patterns of nocturnal enhancement were not detected for bradycardia to injury threat. This effect is surprising given that RA and nocturnal influence in ethology and behavioral ecology are both closely associated with defense against predators and aggressive conspecifics, i.e., prototypical injury threats (Stankowich and Blumstein 2005; Kronfeld‐Schor et al. 2013). Selective results with infection threat stimuli may bolster the idea that the nocturnal period specifically amplifies the attentional component of RA. Infection threats are generally more ambiguous in signaling their “threat potential” than injury threats (Schaller 2016; Fink et al. 2018). Infection threats therefore require greater attentional engagement, cognitive analysis, and stronger bradycardia, which are processes suggestive of RA (Van Hooff et al. 2013, 2014; Ruiz‐Padial et al. 2017).

Previous studies have demonstrated nocturnal enhancement of threat measures that may not reflect RA, namely subjective emotion measures (Li et al. 2015; Emens et al. 2020; Smyth et al. 2009) or physiological response to loud noise (Miller and Gronfier 2006; Pace‐Schott et al. 2014). Extending on this work, we show that the nocturnal period enhances RA towards image stimuli that were chosen to elicit RA. Threat image stimuli possess “aesthetical distance” and activate passive information‐gathering as opposed to active coping such as fight/flight to loud noise (Lang et al. 1993, 2000). Based on our present cardiac findings, we speculate that attention is prolonged, and in some cases amplified (i.e., in high‐anxiety individuals), to prevent hidden threats from being missed at night—since nocturnal darkness concealed visual dangers in the evolutionary environment. Sustained vigilance, manifesting as prolonged threat bradycardia, likely represents a “better safe than sorry” strategy favored by natural selection in uncertain threat contexts such as the nocturnal period (Stein and Nesse 2011).

Importantly, the bradycardia responses observed in the current data are likely reflective of threat‐evoked orienting as opposed to other defensive responses. Bradycardia can also emerge in later parts of the defense cascade within so‐called “freezing” and “tonic immobility” states when threats are either closer, escapable, or less avoidable (Roelofs 2017; Hagenaars et al. 2014; Löw et al. 2015). Since our bradycardia response occurs right after detecting a novel threat stimulus, the response likely taps into RA as opposed to more active/preparatory defensive strategies that occur later towards a dynamic or approaching cue.

Contrary to hypotheses, we found no evidence for nocturnal enhancement of threat‐induced risk aversion after either threat type. Surprisingly, we instead found evidence that the daytime enhances the effects of injury threat (but not infection threat) on increasing risk aversion. This is consistent with prior work where injury threat‐induced risk aversion presumably occurs during daytime procedures (Clark et al. 2012). The daytime enhancement of risk aversion only emerged in situations of risk‐with‐no‐ambiguity. These findings point to specific threat‐related risk averse behavior when individuals have more rather than less information about their decision options. The fact that we observed heightened risk aversion to threats during the daytime is aligned with a diurnal shift in risk taking behavior, where individuals are less likely to take risks earlier rather than later in the day (Li et al. 2020; Bedder et al. 2023).

There was also evidence of enhanced risk aversion following infection threat, consistent with prior work (Kasheer and Nam 2021); that effect did not differ by time‐of‐day. Infection threat's effect on increasing risk aversion was specific to risk‐with‐ambiguity trials, when there was a partial lack of information about outcome probabilities. That specificity may be owed to infection threat and ambiguity trials both reflecting a great deal of uncertainty; as noted earlier, infection threats are believed to be more ambiguous and less direct than injury threats. Such ambiguity processing may prime ambiguity processing in the decisional part of the trial, thus leading to stronger ambiguity aversion.

The fact that the nocturnal period did not enhance risk aversion like it enhanced attention vis‐a‐vis threat‐induced bradycardia is intriguing. This lack of effect may be owed to our focus on value‐based decisions as opposed to decisions about defensive action, which is a primary decisional outcome in RA theories (Blanchard et al. 2001; Gladwin et al. 2016). While individuals are possibly more cautious at night when making decisions about defensive behavior, such as whether to flee, freeze, etc., it is possible that such nocturnal caution does not emerge in choices about seeking out rewards. Ecological theory suggests that when encountering a threat in a dangerous context, an animal's decision to seek out risky rewards (high calorie food amidst predator) may not always be decreased (Lima and Dill 1990). The animal may instead choose to increase or decrease reward‐seeking such as foraging depending on a host of environmental variables such as reward deficit and distance from the threat. As an additional interpretation, threat‐induced risk aversion may be limited to the day period because the daytime is associated with heightened subjective energy (Wood and Magnello 1992), and such heightened cognitive arousal is possibly required for threat‐induced risk aversion. Risk aversion has been shown to rely on complex information processing and the related expenditure of mental energy (Winecoff and Huettel 2017). Furthermore, threat effects on decision‐making also likely require cognitive effort and mental arousal. The weaker injury threat‐induced risk aversion at night could therefore be attributed to the weaker cognitive control and reduced cognitive arousal that accompany the nighttime (Horne 2012). At the very least, our results suggest that threat‐induced bradycardia and threat‐induced risk aversion (about rewards) are separate dimensions of RA that are differentially impacted by context. Indeed, we found no evidence that threat‐induced bradycardia and threat‐induced risk aversion are associated at the within‐ or between‐person levels (see Supporting Information for results)—suggesting that these RA metrics tap into separable and uncorrelated aspects of the RA process. Future studies are needed to further interrogate our speculations and to clarify differences between physiological and behavioral indices of RA.

An inter‐person relationship between RA and anxiety symptoms was found only for infection‐threat induced bradycardia at night. That is, individuals with elevated threat‐induced bradycardia at night exhibited an increased risk for an anxiety disorder, estimated with the DASS‐21; threat‐induced bradycardia measured during the day did not signal risk for anxiety disorder. This result suggests that RA's attentional aspect, when measured at night, may better identify anxiety risk. Other RA measures—bradycardia to injury threat and all metrics of threat‐induced risk aversion—were unrelated to risk for anxiety disorder via the DASS‐21.

Anxiety phenotypes being expressed by anxiogenic contexts is consistent with some prior human studies (Grillon 2002). In one report, panic disorder patients showed heightened startle responding towards unpredictable threat but not towards predictable threat (Grillon 2008). Especially relevant to the current findings, other studies report that PTSD patients have exaggerated startle reactivity under darkness (Grillon et al. 1998; Kavaliers and Choleris 2001). Our current findings make a novel contribution to this literature which has largely focused on narrow changes to the immediate environment. It is unclear whether the nocturnal period is special in activating RA‐anxiety linkages or whether any anxiogenic context is sufficient in activating this phenotype. For example, it is possible that our observed nocturnal effects on anxiety are in part driven by darkness‐enhanced RA, which in turn enhanced bradycardia to ambiguous threats (e.g., infection cues). Future studies should inter‐leave the effect of time‐of‐day and light/dark to explore the mediating mechanisms.

Prior research supports heightened RA, including threat‐induced bradycardia, as a core mechanism underlying risk for psychopathology (e.g., anxiety disorders) (Blanchard and Meyza 2019; Schipper et al. 2019; Thayer et al. 2000). Extending on this work, our findings indicate that threat‐induced bradycardia's status as an anxiety phenotype is dependent on the nighttime context. The nighttime context may amplify this measure as an anxiety risk phenotype (Grillon et al. 2019) because the night context signals danger and uncertainty, which in response activates threat reactions. We speculate that clinically anxious individuals might be easier to identify at night because this anxiogenic context activates the RA processes (vigilance, autonomic nervous system activity) at the core of their symptoms (Grillon 2002). These results suggest that heightened average levels of vigilance could signal mental health difficulties, whereas a pattern of non‐habituating vigilance, reflected as reduced within‐person change in cardiac measures, might instead signal an adaptive mechanism for survival. The functional distinctions between average and within‐person measures of RA require clarification in future studies.

The current study simulates important questions about the evolutionary and biological mechanisms that possibly underlie our nocturnal effects. From neuroethological and behavioral ecological perspectives, threat responses are adaptive if they are modified to fit the environment (Kavaliers and Choleris 2001; Gaynor et al. 2019; Blanchard and Blanchard 2008). We posit that the observed diurnal effects reflect an evolutionarily‐rooted reaction to potential darkness—even if darkness is not present. This adaptive heuristic, involving sustained vigilance, could enhance survival in an evolutionary environment where a lack of defense/vigilance is especially costly (Nesse 2005). In other words, the night context may signal to the brain, body, and mind that there is an increased chance of uncertain danger, given that nocturnal darkness (and related nocturnal features) was a likely selection pressure on the evolution of defensive reactions in humans in our ancestral environments (Kronfeld‐Schor et al. 2013). The fact that nocturnal effects were observed under illuminated conditions suggests that the diurnal shifts in RA (and RA‐anxiety relationships) could be circadian or endogenous in nature—such that acute ambient darkness is not required to drive the nocturnal modulation of defensive responses. Diurnal change in RA in the real world is likely regulated by a complex interaction between internal mechanisms (internal “clocks” in hypothalamus) and external mechanisms (external “zeitgebers” like darkness) (Sollars and Pickard 2015). This is in accord with known chronobiological mechanisms that regulate 24‐h cycles in autonomic reactivity, stress, and cognition (Agorastos et al. 2020; Koch et al. 2017). Such speculated mechanisms are adaptive, but if elevated at the “trait” level, they could predispose individuals to psychopathology, which is consistent with the nocturnal period amplifying relations between bradycardia (to infection threat cues) and anxiety symptoms.

The current study has a number of limitations that future research should address to better understand time‐of‐day impacts on human RA. First, the current findings underscore responses to infection threats, as opposed to injury threats, as being especially sensitive to the nighttime period—possibly because infection threats are more ambiguous and need higher RA at night. Future research is required to test this psychological explanation; for example, studies could vary how much of the threat images are occluded or pixelated to test whether ambiguity does indeed account for the nocturnal effects on some threat types versus others. As an alternative explanation, nocturnal effects may have been limited to infection threat because testing occurred in an indoor environment where shared surfaces (e.g., tables) were salient. If the participants were immersed in an outside environment that is associated with predators, for example, nocturnal effects on injury threat could have been more prominent. Future time‐of‐day studies could use virtual reality paradigms to simulate such naturalistic predator environments without sacrificing experimental rigor. Second, testing sessions were illuminated for both time‐of‐day groups. Current findings therefore suggest that nocturnal effects on RA do not require acute environmental darkness. However, our results do not rule out environmental darkness as a mechanism that augments nocturnal effects beyond what is observed in the current data. Future studies should directly test day versus night effects on RA metrics under different lighting conditions such as light versus dark. For example, night versus day effects on RA may be strongest when comparing dark/night conditions with day/light conditions, since the lighting conditions are congruent with the naturalistic settings. That design would better test whether it is darkness that mediates nocturnal effects on RA and RA‐anxiety relationships. Third, our focus on threat‐induced bradycardia and risk aversion is limited because those metrics only partially index RA, which is a complex process that relies on multiple neural, physiological, and cognitive systems. Future studies should simultaneously assess threat‐induced bradycardia alongside brain imaging measures, such as EEG or fMRI responses, that capture defensive circuitry and attentional network activations. Such neurophysiological assessments could be combined with passive image viewing or threat identification performance (e.g., identify image as threat or not) to bolster inferences regarding nocturnal effects on attentional processes. Tonic and phasic threat‐evoked brain activations in these paradigms could be useful to disentangle nocturnal effects on attentional vigilance (tonic) versus attentional orienting (phasic). Fourth, we did not have a design element to mitigate potential confounds of time‐of‐day effects, namely the heightened fatigue that might occur at night versus day. Present findings, however, suggest that mental fatigue is not a robust confound of time‐of‐day effects in the current data. This is because self‐reported feelings of being alert and active—PANAS items overlapping with fatigue—were not significantly different between day and night groups. Furthermore, the observed pattern of heightened attention to threat at night, inferred from threat‐induced bradycardia results, would suggest less (not more) fatigue at night. Future work should use more precise, objective measures of mental and physical fatigue to more rigorously test it as a confound of nighttime effects. Fifth, our metrics of RA behavior (risk aversion) and anxiety symptoms should be expanded upon in future work. Risk aversion, as noted earlier, captures decision‐making about rewards and not about avoiding threats, with the latter being more central in the RA literature (Blanchard et al. 2011). Additional studies could examine time‐of‐day effects on decision‐making about which defensive strategy to employ: freeze, fight, flight, etc. Virtual reality studies that simulate ecological threat environments (e.g., simulated predator) may prove useful in probing dynamic choices related to defensive strategy.

Overall, time‐of‐day is shown to be an important variable to consider in the study of human threat responses and risk for mental health difficulties. The current study also points to a potential diurnal rhythm in threat‐induced bradycardia that is relevant to vulnerability for anxiety disorders. We propose that nocturnal changes in RA physiology to ambiguous threat could represent a context‐bound phenotype that, on the one hand is evolutionarily protective, but on the other hand leads to mental health risk when amplified on average across time. Future research is needed to substantiate the underlying neural and ecological mechanisms of these novel findings. This work lays the foundation for future research that can potentially transform the anecdote of “heightened fear at night” to a scientifically tested phenomenon.

## Author Contributions

**Derek P. Spangler:** conceptualization, investigation, writing – original draft, methodology, validation, visualization, writing – review and editing, formal analysis, project administration, data curation, supervision, resources. **Richa Gautam:** data curation, methodology, software. **Jennifer T. Kubota:** conceptualization, writing – review and editing, methodology. **Jasmin Cloutier:** conceptualization, writing – review and editing, methodology. **Nina Lauharatanahirun:** conceptualization, investigation, visualization, methodology, writing – review and editing, software, resources.

## Funding

The research was supported by laboratory startup funds provided by Penn State University.

## Ethics Statement

The research presented in this paper has been approved by the Penn State Institutional Review Board. All procedures are in accordance with the Declaration of Helsinki.

## Consent

All research participants provided informed consent before completing the study procedures.

## Conflicts of Interest

The authors declare no conflicts of interest.

## Supporting information


**Figure S1:** Infection threat bradycardia as a function of trial and time‐of‐day. The y‐axis depicts mean bradycardia values that were estimated from the multilevel model. Lines represent the simple slopes from the same model. They test mean differences in bradycardia between infection threat and neutral images (i.e., dummy code effects) at specific levels of trial. Slopes for the first (#1), middle (#30), and last (#60) trial are plotted here. Shaded regions depict model‐based 95% CI intervals. In the day group, the pattern of greater bradycardia (peak deceleration) to infection threat versus neutral images attenuated across trials (Trial 1: *B* = 28.75, *p* < 0.0001; Trial 30: *B* = 18.56, *p* < 0.0001; Trial 60: *B* = 8.03, *p* = 0.048). In the night group, the pattern of greater bradycardia to infection threat versus neutral images did not significantly attenuate across trial (Trial 1: *B* = 18.89, *p* < 0.0001; Trial 30: *B* = 19.16, *p* < 0.0001; Trial 60: *B* = 19.43, *p* < 0.0001).
**Figure S2:** Threat‐induced bradycardia with heart rate (HR) scores in beats per minute (bpm). Panel A: Bradycardia time courses during image viewing. Lines reflect time courses in raw HR deceleration scores across participants and images, estimated as smoothed average scores using a LOESS procedure. Panel B: Injury threat bradycardia habituates between blocks, collapsing across time‐of‐day. The bars depict mean bradycardia by image type and blocks, calculated as the average of the peak HR deceleration scores, collapsing across images, time‐of‐day, and participants. The whiskers depict within‐person standard errors. Panel C: Infection threat bradycardia habituates during the day but not at night. Bars depict mean bradycardia by image type, blocks, and time‐of‐day; calculated as the average of peak HR deceleration scores collapsing across images and participants. The whiskers depict within‐person standard errors.
**Table S1:** Multilevel Model: Average Threat‐Induced Bradycardia (without Habituation).
**Table S2:** Multilevel Model: Habituation in Threat‐Induced Bradycardia.
**Table S3:** Multilevel Model: Average Threat‐Induced Risk Aversion (without Habituation).
**Table S4:** Multilevel Model: Habituation in Threat‐Induced Risk Aversion.
**Table S5:** Logistic Regression Models: Associations between Threat Metrics and Anxiety Symptoms (Without Time‐of‐Day).

## Data Availability

The datasets and RStudio scripts for data processing and statistical analysis can be accessed here: https://figshare.com/s/2544bd4d65e74edea4d3. Please direct any questions about the data and/or code to the corresponding author.
