# Supplementary material for: Nocturnal Risk Assessment and Its Association With Anxiety Symptoms
Source: Psychophysiology. 2026 Mar 23;63(3):e70275. doi: 10.1111/psyp.70275 (PMC13009328; doi:10.1111/psyp.70275)
Supplement: Supplementary file 1 — Figure S1: Infection threat bradycardia as a function of trial and time‐of‐day. The y‐axis depicts mean bradycardia values that were estimated from the multilevel model. Lines represent the simple slopes from the same model. They test mean differences in bradycardia between infection threat and neutral images (i.e., dummy code effects) at specific levels of trial. Slopes for the first (#1), middle (#30), and last (#60) trial are plotted here. Shaded regions depict model‐based 95% CI intervals. In the day group, the pattern of greater bradycardia (peak deceleration) to infection threat versus neutral images attenuated across trials (Trial 1: B = 28.75, p < 0.0001; Trial 30: B = 18.56, p < 0.0001; Trial 60: B = 8.03, p = 0.048). In the night group, the pattern of greater bradycardia to infection threat versus neutral images did not significantly attenuate across trial (Trial 1: B = 18.89, p < 0.0001; Trial 30: B = 19.16, p < 0.0001; Trial 60: B = 19.43, p < 0.0001). Figure S2: Threat‐induced bradycardia with heart rate (HR) scores in beats per minute (bpm). Panel A: Bradycardia time courses during image viewing. Lines reflect time courses in raw HR deceleration scores across participants and images, estimated as smoothed average scores using a LOESS procedure. Panel B: Injury threat bradycardia habituates between blocks, collapsing across time‐of‐day. The bars depict mean bradycardia by image type and blocks, calculated as the average of the peak HR deceleration scores, collapsing across images, time‐of‐day, and participants. The whiskers depict within‐person standard errors. Panel C: Infection threat bradycardia habituates during the day but not at night. Bars depict mean bradycardia by image type, blocks, and time‐of‐day; calculated as the average of peak HR deceleration scores collapsing across images and participants. The whiskers depict within‐person standard errors. Table S1: Multilevel Model: Average Threat‐Induced Bradycardia (without Habituation). Table S2: M [file PSYP-63-e70275-s001.docx]

**Supplemental Materials**

**Validating Habituation Effects using All Levels of Trial**

In the Results section, habituation effects were modeled with a *Block* regressor that compared the first half and second half of trials in each condition. Here, we validate those habituation results by modeling RA measures as a function of trial number (i.e., every trial number between 1 and 60) in each condition. This approach tests habituation as a more continuous, linear change in RA across the condition. The trial effects were tested using the same multilevel model structures except *Block* was replaced with a *Trial* (0-59) regressor. Separate models were conducted separately for bradycardia versus risk aversion as dependent measures. The new effects with *Trial* were similar to analogous effects testing *Block* in the Results section, as is summarized below.

**Threat-Induced Bradycardia**. Regarding habituation in bradycardia to injury threat, the effect was validated such that there was a non-significant *Injury vs. Neutral***Trial* interaction (*B* = -0.15, *p* = 0.085, 95% CI [-0.33, 0.02]) having a similar size and direction as the analogous *Injury vs. Neutral***Block* interaction (Fig. 2B). We next turn to the time-of-day effect on habituation in bradycardia to infection threat. This effect was also validated. The *Infection vs. Neutral***Trial*Time-of-Day* interaction was statistically significant (*B* = 0.36, *p* = 0.020, 95% CI [0.06, 0.66]) with the same magnitude and direction as the *Infection vs. Neutral***Block*Time-of-Day* (Fig. 2C). Probing the interaction revealed a significant *Infection vs. Neutral***Trial* interaction in the day (*B* = -0.35, *p* = 0.003, 95% CI [-0.58, -0.12]) but not the night group (*B* = 0.009, *p* = 0.938, 95% CI [-0.22, 0.24]), suggesting that infection threat effects on bradycardia attenuate to become non-significant across trials during the day. At night, the infection threat effect on bradycardia remained elevated and statistically significant across trials (Fig. S1 below).

**Threat-Induced Risk Aversion**. The pattern of habituation in risk aversion was validated. The *Trial* effect on risky choice was statistically significant and negative in direction (*B* = -0.002, *p* = 0.034, 95% CI [-0.004, -0.0002]), like the effect of *Block* on risky choice reported in Table S7.


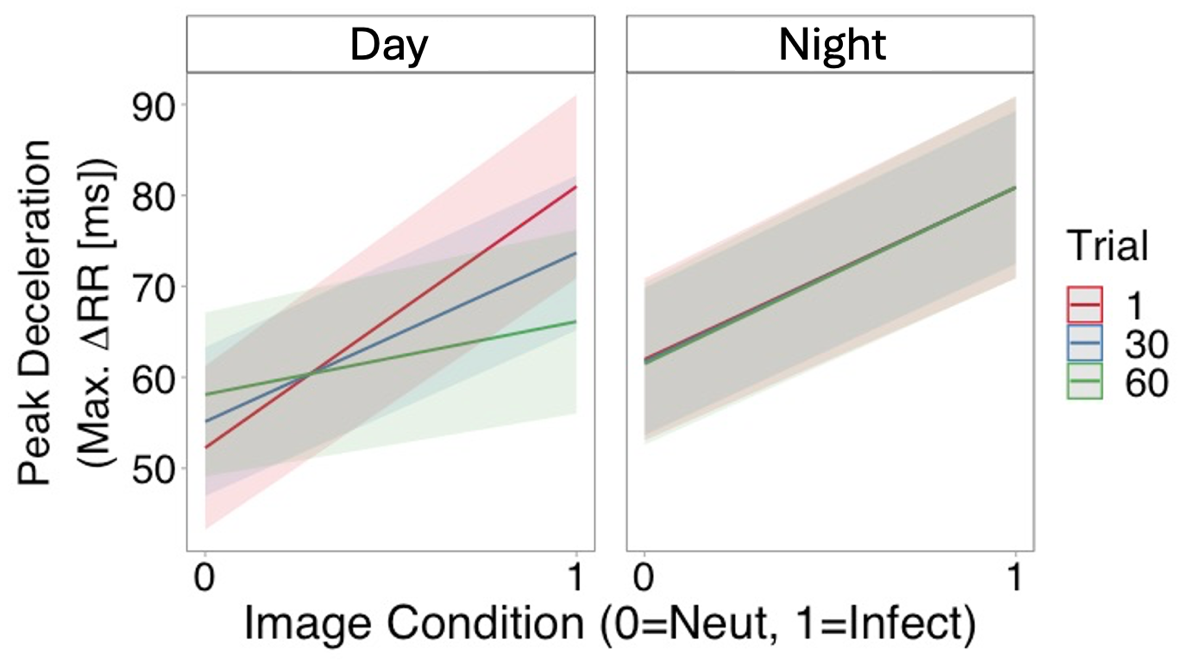


Figure S1. Infection threat bradycardia as a function of trial and time-of-day. The y-axis depicts mean bradycardia values that were estimated from the multilevel model. Lines represent the simple slopes from the same model. They test mean differences in bradycardia between infection threat and neutral images (i.e., dummy code effects) at specific levels of trial. Slopes for the first (#1), middle (#30), and last (#60) trial are plotted here. Shaded regions depict model-based 95% CI intervals. In the day group, the pattern of greater bradycardia (peak deceleration) to infection threat versus neutral images attenuated across trials (Trial 1: *B* = 28.75, *p* < 0.0001; Trial 30: *B* = 18.56, *p* < 0.0001; Trial 60: *B* = 8.03, *p* = 0.048). In the night group, the pattern of greater bradycardia to infection threat versus neutral images did not significantly attenuate across trial (Trial 1: *B* = 18.89, *p* < 0.0001; Trial 30: *B* = 19.16, *p* < 0.0001; Trial 60: *B* = 19.43, *p* < 0.0001).

Abbreviations: Neut = neutral images, Infect = infection threat images.

**Relations between RA components: Bradycardia and risk aversion**

Given differential effects of time-of-day on bradycardia and risk aversion, we sought to better understand the relations between these RA metrics both at the within-person and between-person levels. At the within-level, if threat-induced bradycardia coincides with threat-induced risk aversion, there should be intra-individual associations between condition-level bradycardia scores (average peak RR deceleration) and condition-level risky choice (proportion of risky choices). Using a multilevel regression model with a random intercept of participant, we found no evidence of a within-person association between bradycardia magnitude and risky choice (*B* = 0.00005, *p* = 0.855, 95% CI -0.0005, 0.0006]). We also tested between-person, or inter-person, relationships between average threat-induced bradycardia and average threat-induced risk aversion. Here, we used the same average bradycardia/risk-aversion metrics that were also employed to test the inter-person associations with anxiety. Pearson zero-order correlations did not reveal any significant between-person associations for any of the image type combinations:

Bradycardia to injury - risk aversion to injury: *r* = 0.04, *p* = 0.743, 95% CI [-0.18, 0.24];

Bradycardia to infection - risk aversion to infection: *r* = 0.05, *p* = 0.646, 95% CI [-0.16, 0.26]; Bradycardia to injury - risk aversion to infection: *r* = -0.04, *p* = 0.703, 95% CI [-0.25, 0.17]; Bradycardia to infection - risk aversion to injury: *r* = -0.07, *p* = 0.521, 95% CI [-0.28, 0.14].


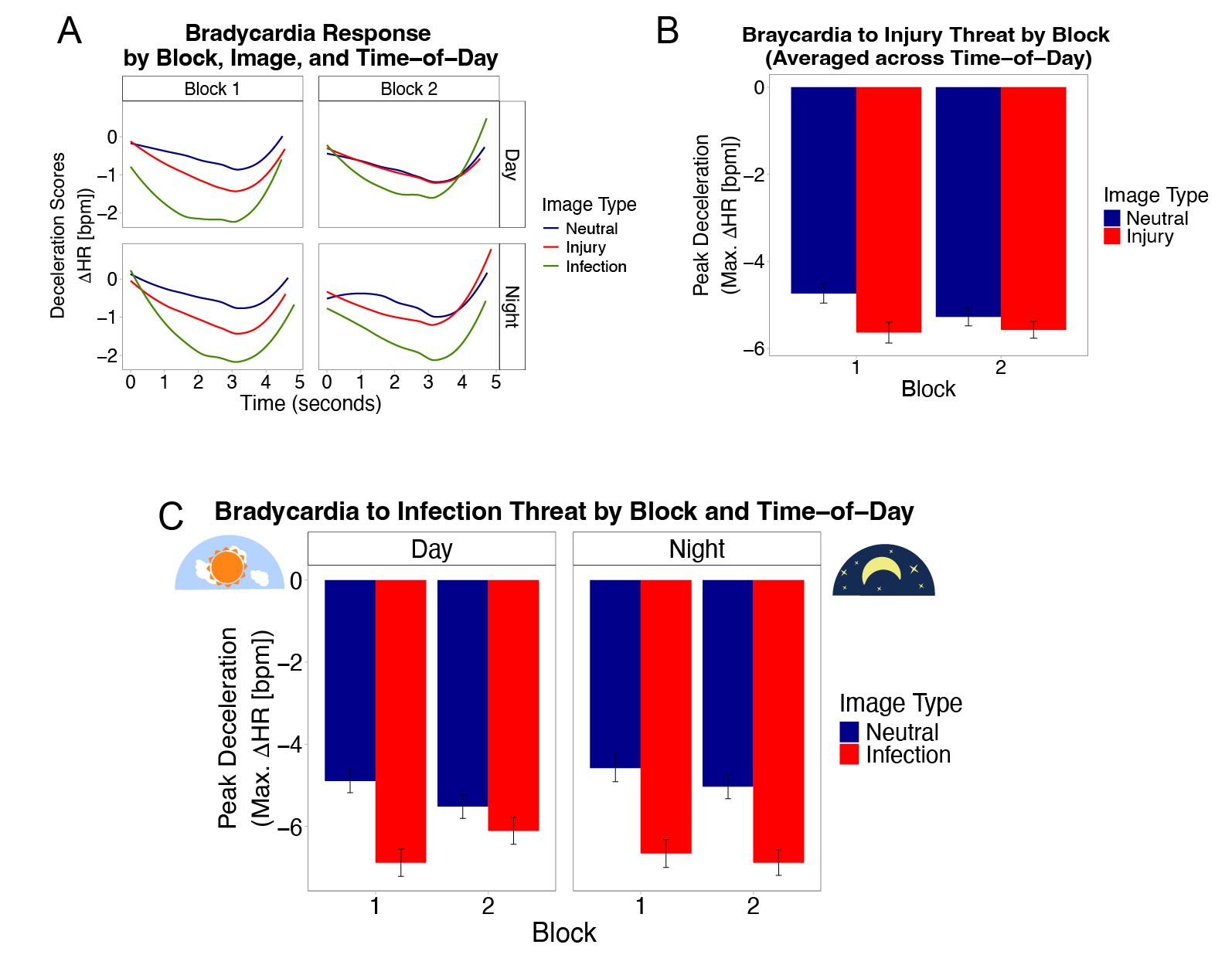


**Figure S2. Threat-induced bradycardia with heart rate (HR) scores in beats per minute (bpm).** ***Panel A*:** **Bradycardia time courses during image viewing**. Notes: Lines reflect time courses in raw HR deceleration scores across participants and images, estimated as smoothed average scores using a LOESS procedure. ***Panel B*:** **Injury threat bradycardia habituates between blocks, collapsing across time-of-day**. The bars depict mean bradycardia by image type and blocks, calculated as the average of the peak HR deceleration scores, collapsing across images, time-of-day, and participants. The whiskers depict within-person standard errors. ***Panel C*:** **Infection threat bradycardia habituates during the day but not at night.** Bars depict mean bradycardia by image type, blocks, and time-of-day; calculated as the average of peak HR deceleration scores collapsing across images and participants. The whiskers depict within-person standard errors.

Table S1. Multilevel Model: Average Threat-Induced Bradycardia (without Habituation)

| **Dependent Measure: Bradycardia** (peak cardiac deceleration during image) | | | | | | | | | | |
| --- | --- | --- | --- | --- | --- | --- | --- | --- | --- | --- |
| ***Fixed Effects*** |  |  |  | |  | |  | |  | |
|  | ***B*** | ***SE*** | ***95% CI: Low*** | ***95% CI: High*** | | ***df*** | | ***p*** | |  |
| Intercept | 54.18 | 4.51 | 45.35 | 63.01 | | 86.99 | | < 0.0001* | |  |
| Injury vs. Neutral | 3.76 | 3.74 | -3.56 | 11.08 | | 86.83 | | 0.317 | |  |
| Infection vs. Neutral | 17.67 | 4.70 | 8.45 | 26.89 | | 87.01 | | 0.0003* | |  |
| Time-of-Day (ToD) | 5.15 | 6.34 | -7.27 | 17.56 | | 86.99 | | 0.419 | |  |
| Injury vs. Neutral*ToD | 2.78 | 5.25 | -7.52 | 13.07 | | 86.80 | | 0.599 | |  |
| Infection vs. Neutral*ToD | 2.19 | 6.62 | -10.77 | 15.16 | | 87.03 | | 0.741 | |  |
|  |  |  |  |  | |  | |  | |  |
| ***Random effects*** |  |  |  | ***Model*** | |  | |  | |  |
|  | ***variance*** |  |  |  | | ***value*** | |  | |  |
| Participant (intercept) | 773.3 |  |  | Deviance | | 180640.7 | |  | |  |
| Injury vs. Neutral (slope) | 400.7 |  |  | AIC | | 180666.7 | |  | |  |
| Infection vs. Neutral (slope) | 752.7 |  |  | ICC | | 0.10 | |  | |  |

A multilevel regression model was fit with maximum likelihood estimation. Unstandardized regression coefficients (*B*) are provided. Dependent Measure: Bradycardia refers to the peak cardiac deceleration (maximum RR interval change score) during the image relative to a pre-image baseline. Fixed Effect Regressors: *Injury vs. Neutral* and *Infection vs. Neutral* are dummy code variables testing differences in bradycardia between threat and neutral images; neutral images were the reference group. These terms quantify average threat-induced bradycardia collapsing across blocks. *Time-of-day* (ToD) was modeled with a binary dummy code and reflected whether procedures were completed during the day (ToD= 0; reference group) or night (ToD=1). *Injury vs. Neutral*ToD* and *Infection vs. Neutral*ToD* represent product-term interactions between the threat dummy variables and time-of-day; these interactions test whether average threat-induced bradycardia differs between day and night collapsing across blocks.

* *p* < 0.05 (two-tailed)

Table S2. Multilevel Model: Habituation in Threat-Induced Bradycardia

| **Dependent Measure: Bradycardia** (peak cardiac deceleration during image) | | | | | | |
| --- | --- | --- | --- | --- | --- | --- |
| ***Fixed Effects*** |  |  |  |  |  |  |
|  | ***B*** | ***SE*** | ***95% CI: Low*** | ***95% CI: High*** | ***df*** | ***p*** |
| Intercept | 51.56 | 5.19 | 41.39 | 61.74 | 86.99 | <0.0001* |
| Injury vs. Neutral | 6.25 | 4.81 | -3.18 | 15.68 | 86.99 | 0.197 |
| Infection vs. Neutral | 26.11 | 5.54 | 15.25 | 36.96 | 86.99 | <0.0001* |
| Block | 5.23 | 3.60 | -1.82 | 12.28 | 87.10 | 0.150 |
| Injury vs. Neutral*Block | -4.97 | 5.53 | -15.80 | 5.87 | 87.06 | 0.371 |
| Infection vs. Neutral*Block | -16.88 | 5.03 | -26.75 | -7.02 | 87.02 | 0.001* |
| Time-of-Day (ToD) | 5.54 | 7.30 | -8.77 | 19.84 | 87.00 | 0.450 |
| ToD*Block | -0.77 | 5.06 | -10.68 | 9.15 | 87.10 | 0.880 |
| Injury vs. Neutral*ToD | 5.15 | 6.77 | -8.11 | 18.42 | 87.00 | 0.448 |
| Infection vs. Neutral*ToD | -4.88 | 7.79 | -20.15 | 10.39 | 87.01 | 0.533 |
| Injury vs. Neutral*Block*ToD | -4.78 | 7.77 | -20.01 | 10.45 | 87.01 | 0.540 |
| Infection vs. Neutral*Block*ToD | 14.17 | 7.08 | 0.30 | 28.04 | 87.10 | 0.048* |
|  |  |  |  |  |  |  |
| ***Random effects*** |  |  |  | ***Model*** |  |  |
|  | ***variance*** |  |  |  | ***value*** |  |
| Participant (intercept) | 961.6 |  |  | Deviance | 180580.3 |  |
| Injury vs. Neutral (slope) | 602.2 |  |  | AIC | 180648.3 |  |
| Infection vs. Neutral (slope) | 926.4 |  |  | ICC | 0.10 |  |
| Block (slope) | 162.1 |  |  |  |  |  |
| Injury vs. Neutral*Block (slope) | 524.9 |  |  |  |  |  |
| Infection vs. Neutral*Block (slope) | 300.6 |  |  |  |  |  |

A multilevel regression model was fit with maximum likelihood estimation. Unstandardized regression coefficients (*B*) are provided. Dependent Measure: Bradycardia refers to the peak cardiac deceleration (maximum RR interval change score) during the image relative to a pre-image baseline. Fixed Effect Regressors: *Injury vs. Neutral* and *Infection vs. Neutral* are dummy code variables testing differences in bradycardia between threat and neutral images; neutral images were the reference group. These terms quantify threat-induced bradycardia. *Time-of-day* (ToD) was modeled with a binary dummy code and reflected whether procedures were completed during the day (ToD= 0; reference group) or night (ToD=1). *Block* is a dummy code variable testing habituation as the differences in bradycardia between block 1 (=0) and block 2 (=1), with block 1 being coded as the reference group. Of note, *Injury vs. Neutral*Block*ToD* and *Infection vs. Neutral*Block*ToD,* represent product-term interactions between the threat dummy variables, block, and time-of-day; these interactions test whether habituation in threat-induced bradycardia differs between day and night.

* *p* < 0.05 (two-tailed)

Table S3. Multilevel Model: Average Threat-Induced Risk Aversion (without Habituation)

| **Dependent Measure: Risky Choice** (log-odds of risky choice) | | | | | |
| --- | --- | --- | --- | --- | --- |
| ***Fixed Effects*** |  |  |  |  |  |
|  | ***B*** | ***SE*** | ***95% CI: Low*** | ***95% CI: High*** | ***p*** |
| Intercept | -0.12 | 0.15 | -0.41 | 0.17 | 0.431 |
| Injury vs. Neutral | -0.18 | 0.09 | -0.35 | -0.005 | 0.044* |
| Infection vs. Neutral | -0.11 | 0.08 | -0.27 | 0.04 | 0.134 |
| Time-of-Day (ToD) | -0.28 | 0.21 | -0.69 | 0.13 | 0.181 |
| Injury vs. Neutral*ToD | 0.24 | 0.13 | -0.01 | 0.49 | 0.057 |
| Infection vs. Neutral*ToD | -0.02 | 0.11 | -0.24 | 0.19 | 0.835 |
|  |  |  |  |  |  |
| ***Random effects*** |  |  |  | ***Model*** |  |
|  | ***variance*** |  |  |  | ***value*** |
| Participant (intercept) | 0.85 |  |  | Deviance | 19626.6 |
| Injury vs. Neutral (slope) | 0.18 |  |  | AIC | 19650.6 |
| Infection vs. Neutral (slope) | 0.10 |  |  |  |  |

A logistic multilevel regression model was fit with maximum likelihood estimation. Unstandardized regression coefficients (*B*) are provided. Dependent Measure: The log-odds ratio of risky choice (i.e., degree of risk aversion). Fixed Effect Regressors*:* *Injury vs. Neutral* and *Infection vs. Neutral* are dummy code variables testing differences in the odds of risky choice (i.e., degree of risk aversion) between threat and neutral images; neutral images were the reference group. These terms quantify average threat-induced risk aversion collapsing across blocks. *Time-of-day* (ToD) was modeled with a binary dummy code and reflected whether procedures were completed during the day (ToD= 0; reference group) or night (ToD=1). *Injury vs. Neutral*ToD* and *Infection vs. Neutral*ToD* represent product interactions between the threat dummy variables and time-of-day; these interactions test whether average threat-induced risk aversion differs between day and night collapsing across blocks.

* *p* < 0.05 (two-tailed)

Table S4. Multilevel Model: Habituation in Threat-Induced Risk Aversion

| **Dependent Measure: Risky Choice** (log-odds of risky choice) | | | | | | | | |
| --- | --- | --- | --- | --- | --- | --- | --- | --- |
| ***Fixed Effects*** |  |  |  | |  | |  | |
|  | ***B*** | ***SE*** | ***95% CI: Low*** | | ***95% CI: High*** | | ***p*** | |
| Intercept | -0.04 | 0.15 | -0.33 | | 0.26 | | 0.810 | |
| Injury vs. Neutral | -0.22 | 0.08 | -0.38 | | -0.05 | | 0.010* | |
| Infection vs. Neutral | -0.17 | 0.08 | -0.33 | | -0.001 | | 0.048* | |
| Block | -0.16 | 0.08 | -0.32 | | 0.01 | | 0.061 | |
| Injury vs. Neutral*Block | 0.09 | 0.12 | -0.14 | | 0.32 | | 0.447 | |
| Infection vs. Neutral*Block | 0.10 | 0.12 | -0.14 | | 0.33 | | 0.413 | |
| Time-of-Day (ToD) | -0.33 | 0.21 | -0.75 | | 0.09 | | 0.120 | |
| ToD*Block | 0.12 | 0.12 | -0.12 | | 0.35 | | 0.326 | |
| Injury vs. Neutral*ToD | 0.34 | 0.12 | 0.10 | | 0.57 | | 0.005* | |
| Infection vs. Neutral*ToD | -0.01 | 0.12 | -0.24 | | 0.23 | | 0.961 | |
| Injury vs. Neutral*Block*ToD | -0.22 | 0.17 | -0.55 | | 0.11 | | 0.187 | |
| Infection vs. Neutral*Block*ToD | -0.03 | 0.17 | -0.36 | | 0.30 | | 0.856 | |
|  |  |  |  | |  | |  | |
| ***Random effects*** |  |  |  | | ***Model*** | |  | |
|  | ***variance*** |  |  | |  | | ***value*** | |
| Participant (intercept) | 0.84 |  |  | Deviance | | 19656.6 | |  |
| Injury vs. Neutral (slope)◊ | 0.14 |  |  | AIC | | 19682.5 | |  |
| Infection vs. Neutral (slope) ◊ | 0.06 |  |  | |  | |  | |
| Block (slope) ◊ | 0.03 |  |  | |  | |  | |
| Injury vs. Neutral*Block (slope) ◊ | 0.03 |  |  | |  | |  | |
| Infection vs. Neutral*Block (slope) ◊ | 0.06 |  |  | |  | |  | |

A logistic multilevel regression model was fit with maximum likelihood estimation. Unstandardized regression coefficients (*B*) are provided. Dependent Measure: The log-odds ratio of risky choice (i.e., degree of risk aversion). Fixed Effect Regressors: *Injury vs. Neutral* and *Infection vs. Neutral* are dummy code variables testing differences in odds of risky choice (i.e., degree of risk aversion) between threat and neutral images; neutral images were the reference group. These terms quantify average threat-induced risk aversion. *Time-of-day* (ToD) was modeled with a binary dummy code and reflected whether procedures were completed during the day (ToD= 0; reference group) or night (ToD=1). *Block* is a dummy code variable testing habituation as the differences in risky choice between block 1 (Block=0) and block 2 (Block=1), with block 1 being coded as the reference group. Of note*, Injury vs. Neutral*Block*ToD* and *Infection vs. Neutral*Block*ToD,* represent product-term interactions between the threat dummy variables, block, and time-of-day; these interactions test whether habituation in threat-induced aversion differs between day and night.

* *p* < 0.05 (two-tailed)

◊ Random effects are from full model that had convergence issues and are provided for completeness. All other effects come from random intercept model without random slopes.

Table S5. Logistic Regression Models: Associations between Threat Metrics and Anxiety Symptoms (Without Time-of-Day)

|  | **Bradycardia Models** | | | | | | | | | | |  |  |
| --- | --- | --- | --- | --- | --- | --- | --- | --- | --- | --- | --- | --- | --- |
|  | **Injury Threat** (Model Fit: AIC = 116.93) | | | | |  | **Infection Threat** (Model Fit: AIC = 118.11) | | | | | | |
|  |  |  |  |  |  |  |  |  |  |  |  | |  |
|  | ***B*** | ***SE*** | ***95% CI: Low*** | ***95% CI: High*** | ***p*** |  | ***B*** | ***SE*** | ***95% CI: Low*** | ***95% CI: High*** | ***p*** | |  |
| Intercept | -0.58 | 0.23 | -1.05 | -0.13 | 0.013* |  | -0.66 | 0.27 | -1.21 | -0.15 | 0.014* | |  |
| Threat-Induced Bradycardia | 0.01 | 0.01 | -0.003 | 0.03 | 0.116 |  | 0.01 | 0.01 | -0.01 | 0.02 | 0.245 | |  |
|  |  |  |  |  |  |  |  |  |  |  |  | |  |
|  | **Risk Aversion Models** | | | | | | | | | | |  |  |
|  | **Injury Threat** (Model Fit: AIC = 119.35) | | | | |  | **Infection Threat** (Model Fit: AIC = 119.33) | | | | | | |
|  |  | | | | |  |  | | | | | | |
|  | ***B*** | ***SE*** | ***95% CI: Low*** | ***95% CI: High*** | ***p*** |  | ***B*** | ***SE*** | ***95% CI: Low*** | ***95% CI: High*** | ***p*** | |  |
| Intercept | -0.50 | 0.22 | -0.95 | -0.07 | 0.024* |  | -0.52 | 0.23 | -0.98 | -0.07 | 0.025* | |  |
| Threat-Induced Risk Aversion | -0.65 | 1.73 | -4.12 | 2.79 | 0.706 |  | -0.79 | 1.99 | -4.78 | 3.12 | 0.691 | |  |

All logistic regression models are conducted on the full sample (N=87) collapsing across time-of-day group. In each model, the dependent measure is the log-odds of membership in the above-threshold anxiety group, signaling heightened risk for clinical anxiety. *Threat-Induced Bradycardia* is the average RR deceleration score to either injury threat (first column) or infection threat (second column) images subtracting out average RR deceleration to neutral images. *Threat-Induced Risk Aversion* is the average probability of risky choice to either injury threat (first column) or infection threat (second column) images subtracting out the average probability of risky choice to neutral images.

* *p* < 0.05 (two-tailed)
